# Patient preferences for pancreatic cancer treatment (PERSEUS): a multicenter discrete choice experiment

**DOI:** 10.1186/s12955-025-02440-5

**Published:** 2025-12-24

**Authors:** Marjolein F. Lansbergen, Ian P. Smith, Evelien N. van Alphen, Simone Augustinus, Ilse J. M. Fransen, Johanna W. Wilmink, Marc G. Besselink, I. Quintus. Molenaar, Marjolein Y. V. Homs, Ignace H. J. T. de Hingh, Bert. A. Bonsing, Judith de Vos – Geelen, Brigitte C. M. Haberkorn, Pauline A. J. Vissers, Pythia T. Nieuwkerk, Maarten F. Bijlsma, Geert W. J. Frederix, Hanneke W. M. van Laarhoven

**Affiliations:** 1 Department of Medical Oncology, Amsterdam UMC, Location University of Amsterdam, De Boelelaan 1117, 1081 HV Amsterdam, The Netherlands; 2 Amsterdam UMC, Location University of Amsterdam, Laboratory of Experimental Oncology and Radiobiology, Amsterdam, The Netherlands; 3 Cancer Center Amsterdam, Amsterdam, The Netherlands; 4 Julius Center for Health Sciences and Primary Care, University Medical Center Utrecht, Utrecht, The Netherlands; 5 Department of Surgery, Amsterdam UMC, Location University of Amsterdam, Amsterdam, The Netherlands; 6 Department of Surgery, Regional Academic Cancer Center Utrecht, University Medical Center Utrecht, University of Utrecht, Utrecht, The Netherlands; 7 Department of Medical Oncology, Erasmus MC Cancer Institute, Rotterdam, The Netherlands; 8 Department of Surgery, Catharina Hospital, Eindhoven, The Netherlands; 9 Department of Epidemiology, GROW-School for Oncology and Developmental Biology, Maastricht University, Maastricht, The Netherlands; 10 Department of Surgery, Leiden University Medical Center, Leiden, The Netherlands; 11 Divison Medical Oncology, Department Internal Medicine, GROW – Research Institute for Oncology & Reproduction, Maastricht University Medical Centre, Maastricht, The Netherlands; 12 Department of Medical Oncology, Maasstad Hospital, Rotterdam, The Netherlands; 13 Department of Research and Development, Netherlands Comprehensive Cancer Organisation (IKNL), Utrecht, The Netherlands; 14 Department of Surgery, Radboud University Medical Center, Nijmegen, The Netherlands; 15 Department of Medical Psychology, Amsterdam UMC, Location University of Amsterdam, Amsterdam, The Netherlands; 16 Amsterdam Public Health Research Institute, Amsterdam, The Netherlands

**Keywords:** Pancreatic ductal carcinoma, Patient preference, Quality of life, Adverse drug events, Clinical relevance

## Abstract

**Background:**

Pancreatic cancer has an aggressive nature, and treatment severely impacts patients’ quality of life. There is limited understanding how patients weigh treatment benefits against side effects, which hampers the development of patient-centered care and shared decision-making programs.

**Methods:**

Two discrete-choice surveys were designed: one comprising pancreatic cancer patients with (borderline) resectable disease (early-stage disease), and one including patients with non-resectable or metastatic disease (late-stage disease). Relevant criteria for describing treatments were identified by literature review and validated through patient and expert interviews. Selected criteria were likelihood of adverse events causing hospitalization, impact on daily functioning, gastrointestinal symptoms, life expectancy and frequency of hospital visits. Interim analysis was executed after 109 inclusions, optimizing the choice task combinations. Patients were recruited from a local center and a nationwide questionnaire project.

**Results:**

Overall, 428 surveys were sent out and 53% of the participants answered at least one choice task. This included 165 participants with early-stage disease and 62 participants with late-stage disease. Most participants had treatment experience before completing the survey. For both disease stages, participants had a significant preference for the treatment options instead of receiving best supportive care only, although there was significant heterogeneity for this preference among the participants. Life expectancy was the most important treatment characteristic of the pre-selected criteria.

**Conclusions:**

Pancreatic cancer patients, both with early-stage and late-stage disease, choose for anti-cancer treatment over best supportive care and value life expectancy as the most important treatment attribute, although significant differences exist between patients.

**Supplementary Information:**

The online version contains supplementary material available at 10.1186/s12955-025-02440-5.

## Highlights

- Despite the immense negative impact of pancreatic cancer on patients’ physical health, life expectancy and health-related quality of life, there is few research into patients’ preferences for treatment and how patients weight treatment side effects and benefits.
- A discrete-choice experiment into treatment preferences of patients in both early-stage and late-stage treatment settings showed a significant preference for life-extending treatment in both patient groups. A life expectancy versus quality of life trade-off assessment was performed.
- In both patient groups was significant heterogeneity in preferences between the respondents, emphasizing the importance of shared decision-making in treatment of early- and late-stage pancreatic cancer.

**Supplementary Information:**

The online version contains supplementary material available at 10.1186/s12955-025-02440-5.

## Introduction

Pancreatic cancer has a dismal prognosis and its incidence is rising. In 2022 in the Netherlands, the incidence was 2835 new cases and the one-year survival rate was 28%. Consequently, pancreatic cancer has the poorest outlook of all cancer types, which hardly improved over recent years [1]. In addition to the limited life expectancy that patients are facing, pancreatic cancer has a large negative impact on quality of life. In several studies reporting health-related quality of life data, patients showed a low general health, with symptoms such as pain, appetite loss, insomnia, depression and anxiety [2–4], and performed worse compared to patients with other cancer types [5].

The majority of the patients with pancreatic cancer do not receive anti-cancer treatment (46–69% of all patients and 57–76% of patients with metastasized pancreatic cancer) [6–11]. Most patients who receive anti-cancer treatment, get systemic therapy, sometimes in combination with surgery when having resectable disease without metastases [12–18]. Despite recent improvements [12, 17], survival rates of patients outside trial populations remain low, ranging from 15.3 to 31.5 months in patients undergoing resection [6, 19–21] and from 6.6 to 11 months in FOLFIRINOX-treated patients with metastatic disease [22–25]. The systemic therapies cause side effects which may further decrease the quality of life of pancreatic cancer patients, such as nausea, diarrhea, fatigue, neutropenia, leukopenia and thrombocytopenia [13, 18, 26].

The side effects caused by anti-tumor treatments combined with the limited increase in life expectancy added by these treatments lead to the question whether current pancreatic cancer treatments are acceptable and worthwhile from a patient perspective. While existing guidelines outline the most effective treatment strategies [27, 28], no standard exists for what constitutes an acceptable balance of survival and quality of life from the patient’s perspective. A recent Delphi study showed which patient-reported outcomes (PROs) are considered to be the most important to patients and healthcare professionals, but this study did not define which changes in PROs as treatment effects are acceptable to patients [4]. There is few literature on pancreatic cancer patients’ preferences. In one study, patients with resectable disease were offered a choice between resection and chemotherapy, and 97% of the participants preferred the potential curative resection over palliative chemotherapy [29]. Although this study stressed the importance of hope for cure, still information is lacking on how patients weighed general quality of life aspects and chemotherapy side effects against life extension by the treatment, particularly for patients with non-resectable and metastatic disease.

Thus, due to the severity of both the disease and the treatment options, more insight is needed into which impact on quality of life is acceptable to pancreatic cancer patients. Therefore, the primary objective of this study was to identify and quantify patient preferences for care, to assess the relative importance of different attributes of care, and to understand the trade-offs for side effects that patients may be willing to accept for life extension.

## Methods

### Preference assessment tool

**Fig. 1:**
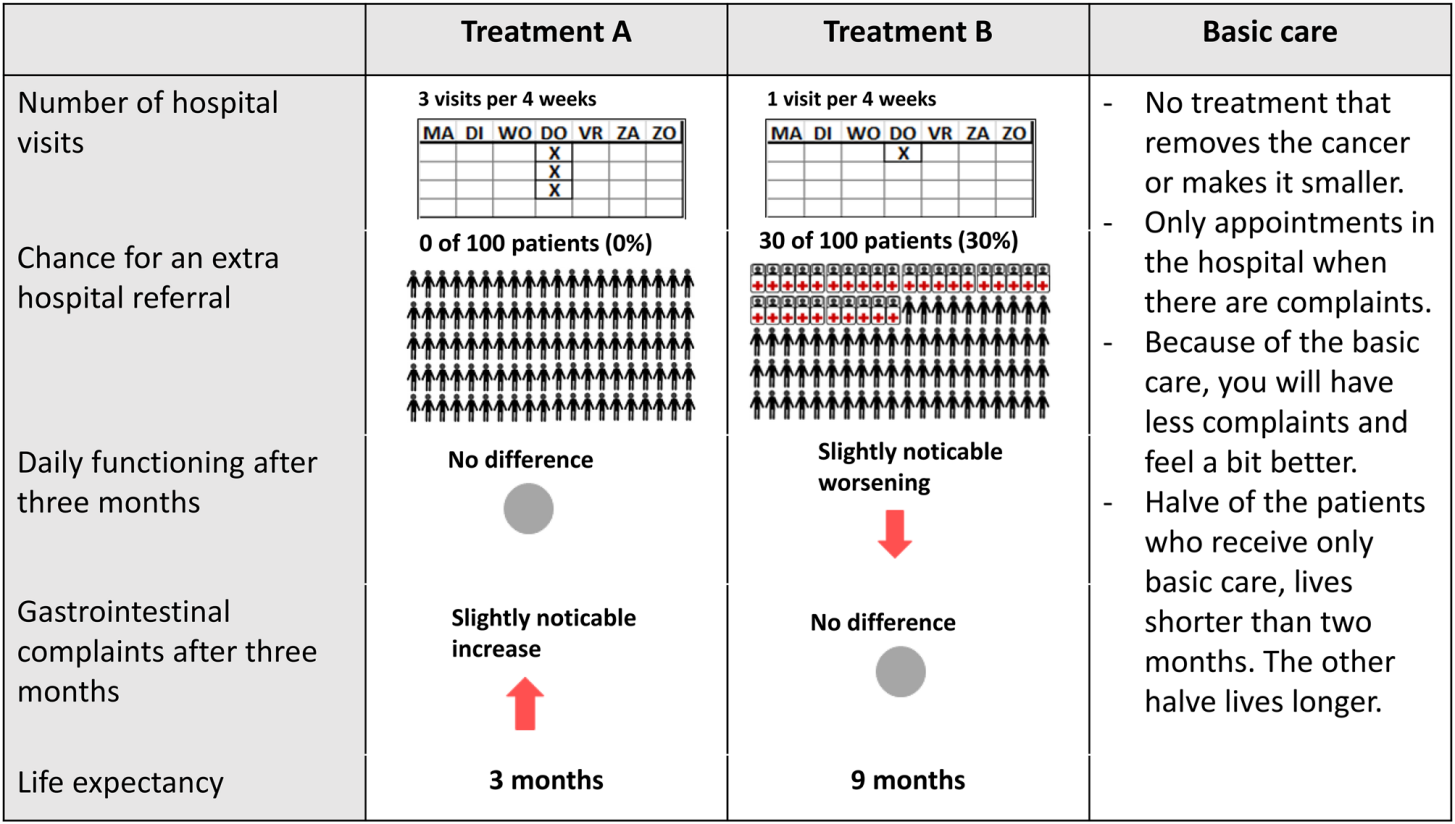
Example choice task in the late-stage disease setting. Participants are asked to choose between two fictional treatment options and a basic care option. Per attribute, the levels are textually and graphically presented

A common and often used method to weigh the importance of specific treatment attributes relative to each other is the discrete choice experiment (DCE) [30]. Background information on DCE is provided in the Supplementary Material2. In a DCE, participants are asked to choose between multiple treatment options described with a selection of *attributes*, treatment characteristics, e.g. the side effects caused by the treatment or the way of administration. Attributes can have various *levels*, e.g.: mild vs. moderate side-effects. In this study, respondents were presented with choice tasks consisting of two fictional treatment options and were asked to choose the most preferred treatment. It was also possible to choose ‘basic care’ which was a representation of best supportive care (BSC) for pancreatic cancer patients, described as symptom-relieving but not life-extending care. An example of a DCE choice task used in this study can be found in Fig. 1. This study had a cross-sectional design, and participants received one survey that had multiple choice tasks. All steps conducted in the design of the survey and the analysis of the results are shown in Supplementary Figure 1.

### Determination of attributes and levels

The DCE was conducted in two patient groups, one including patients with early-stage disease and one including patients with late-stage disease. To select attributes, a literature review was executed (Supplementary Methods, Supplementary Table 1) and subsequently attributes were discussed with eight experts in the field of pancreatic cancer (e.g. oncologists, surgeons) and experts in the field of DCE (e.g. researchers and a medical psychologist). Five attributes were identified as being relevant for patients when choosing their treatment: Daily functioning; Gastrointestinal complaints; Overall disease-related survival; Chance of additional hospitalization; and Number of hospital visits required to receive treatment. These attributes were the same for early- and late-stage disease survey settings, although their levels varied between the settings. In the Supplementary Material 2, the attribute level determination was described. The final attributes and levels used in the choice tasks are shown in Tables 1 and 2.

**Table 1:** Treatment attributes and levels for early-stage disease setting questionnaires

Attribute	Levels (scale)	Levels (meaning)
Daily functioning at three months after surgery	−9 to 9 points	Daily functioning is the same as before surgery
	−9 to −18 points	Daily functioning has slightly worsened compared to before surgery
	−18 to −27 points	Daily functioning has clearly worsened compared to before surgery
Gastrointestinal complaints at three months after surgery	−5 to −10 points	Gastrointestinal complaints are slightly decreased compared to before surgery
	−5 to 5 points	Gastrointestinal complaints are the same as before surgery
	5 to 10 points	Gastrointestinal complaints are slightly worsened compared to before surgery
Life expectancy after treatment start	6 months	People who chose this intervention, live until six months after surgery
	12 months	People who chose this intervention, live until twelve months after surgery
	18 months	People who chose this intervention, live until eighteen months after surgery
	24 months	People who chose this intervention, live until twenty-four months after surgery
Adverse events that cause hospitalization	0%	None of 100 patients (0%) gets serious complaints because of the intervention and needs extra hospitalization
	30%	30 of 100 patients (30%) gets serious complaints because of the intervention and needs extra hospitalization
	60%	60 of 100 patients (60%) gets serious complaints because of the intervention and needs extra hospitalization
Number of hospital visits	10 days	Hospital stay of ten days to undergo surgery
	10 days +6 visits	Hospital stay of ten days to undergo surgery, afterwards six visits to receive chemotherapy, during three months
	10 days +12 visits	Hospital stay of ten days to undergo surgery, afterwards twelve visits to receive chemotherapy, during six months

Selected attributes and levels including their meaning as presented to the participants for the early-stage disease setting

**Table 2:** Treatment attributes and levels for late-stage disease setting questionnaires

Attribute	Levels (scale)	Levels (meaning)
Daily functioning at three months after start chemotherapy	−9 to −18 points	Daily functioning has slightly worsened compared to before the first cycle of chemotherapy
	−9 to 9 points	Daily functioning is the same as before the first cycle of chemotherapy
	9 to 18 points	Daily functioning has slightly increased compared to before the first cycle of chemotherapy
Gastrointestinal complaints at three months after start chemotherapy	−10 to −15 points	Gastrointestinal complaints are clearly decreased compared to before the first cycle of chemotherapy
	−5 to −10 points	Gastrointestinal complaints are slightly decreased compared to before the first cycle of chemotherapy
	−5 to 5 points	Gastrointestinal complaints are the same as before the first cycle of chemotherapy
	5 to 10 points	Gastrointestinal complaints are slightly worsened compared to before the first cycle of chemotherapy
Life expectancy after treatment start	3 months	People who chose this intervention, live until three months after the first cycle of chemotherapy
	6 months	People who chose this intervention, live until six months after the first cycle of chemotherapy
	9 months	People who chose this intervention, live until nine months after the first cycle of chemotherapy
	12 months	People who chose this intervention, live until twelve months after the first cycle of chemotherapy
Adverse events that cause hospitalization	0%	None of 100 patients (0%) gets serious complaints because of the intervention and needs extra hospitalization
	15%	15 of 100 patients (15%) gets serious complaints because of the intervention and needs extra hospitalization
	30%	30 of 100 patients (30%) gets serious complaints because of the intervention and needs extra hospitalization
Number of hospital visits	1 visit per 4 weeks	One visit each four weeks to receive chemotherapy
	2 visit per 4 weeks	Two visits each four weeks (one visit every two weeks) to receive chemotherapy
	3 visit per 4 weeks	Three visits each four weeks (one visit every week for three weeks and then a week no visit) to receive chemotherapy

Selected attributes and levels including their meaning as presented to the participants for the late-stage disease setting

### DCE design development

The experimental design of the DCE was obtained from NGene [31]. According to international guidelines, a D-efficient optimized design was used to maximize efficiency of results in the main survey [32]. By this method, the most relevant combinations of choice tasks were selected to obtain a maximal amount of information needing a minimal amount of choice tasks. The initial design used patients’ and experts’ ranking of the included attributes and levels as preliminary priors for the design (Supplementary Material 2). At first instance we estimated a D-efficient multinomial logit (MNL) fractional factorial main effects design, generating 33 (early-stage disease) and 36 (late-stage disease) choice sets using Ngene software. To reduce the number of questions that participants had to complete, the full design was blocked into three versions of the survey [33]. All attribute levels appeared with equal frequency in each version. The questionnaire was tested by both involved and non-involved cancer-treating physicians, and later by lay people, and the questionnaire was adapted based on their feedback. The first four included patients also provided feedback by phone after completing the questionnaire, not prompting any new changes. After the first 109 patients were included (45 late-stage and 64 early-stage setting), the questionnaire design was optimized for efficiency (Supplementary Table 3 and 4 showing results interim analysis). A conditional logit model was estimated to produce separate prior coefficients for patients. These priors were used to optimize the DCE design for the second phase of patient inclusion.

### The DCE survey

The questionnaire started with questions regarding demographic information and treatment (Supplementary Material 1 (translated to English)). Before starting the choice tasks, respondents received additional information describing relevant details about the different attributes and levels. Also, two questions were added to test the understandability of the attributes and levels. Next, the choice tasks were presented to the participants. Because of the blocked design, a third of all fictional treatment combinations was asked to the participants: 11 in the early-stage setting and 12 in the late-stage setting. An example of a choice task used in this study is presented in Fig. 1.

### Study population and recruitment

**Fig. 2:**
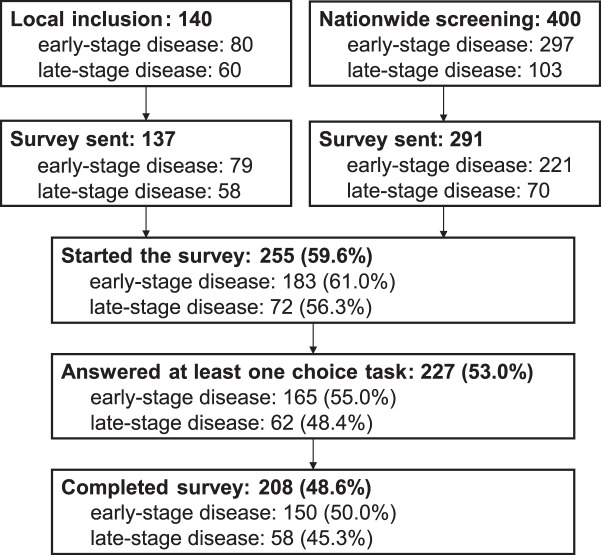
Flow chart of patient screening and inclusion. 428 surveys were sent; the presented percentages are proportions of this number. All patients who completed at least one choice task were included in the analysis. PACAP (pancreatic cancer project) is a nationwide cohort study and active PACAP participants were approached to participate in the discrete-choice experiment. 164 patients with early-stage disease and 63 patients with late-stage disease answered at least on choice task, but one patient with late-stage disease received a survey for early-stage disease, resulting these final inclusion numbers

Eligible respondents were diagnosed with pancreatic cancer (pancreatic ductal adenocarcinoma), at least 18 years of age and were able to understand written Dutch. Both treatment-naïve patients or those who underwent anti-cancer treatment in the past could be included. Patients with early-stage disease had either upfront resectable, borderline resectable or locally advanced disease or underwent a resection in the past. In case of locally advanced disease, patients were considered curative setting (early-stage disease) if their treatment was with curative intent according to the patient record. Patients with unresectable or metastatic disease, or a disease recurrence after resection, had late-stage disease. Patients were recruited by two routes. First, patients were included at the Amsterdam UMC HPB outpatient clinic. After giving informed consent, patients received a survey by e-mail, except when they wished to receive a paper version (19/137, 13.9%). Additionally, patients were recruited from the Dutch PACAP-PROMs questionnaire project [3]. This is a nationwide, multicenter cohort where participants receive quality-of-life questionnaires at a regular interval. Active participants in the PACAP-PROMs were approached by paper mail with the questionnaire, the informed consent form and an information letter. In total, patients were included from 25 centers. The treatment setting of these patients was based on the treatment plan as it was registered in the Netherlands Cancer Registry (NCR). Here, the setting was determined as early-stage when the patient had resectable or borderline resectable disease at diagnosis, and as late-stage when the patient had locally advanced or metastatic disease at diagnosis. The flow of inclusion is shown in Fig. 2.

### Sample size

Previous research has shown that in all DCE studies with efficient designs, precision increases at sample sizes greater than 150 and flattens around 300 [32]. Therefore we aimed to include at least 150 participants per treatment setting.

### Attributes assessment

See Supplementary Material 2 for a note on analysis of the baseline characteristics. For each treatment setting, the treatment attributes and levels that were used in the choice tasks to describe the fictive treatment options (as mentioned in Tables 1 and 2) were analyzed separately. The analysis was performed according to international standards for DCE analysis and other researchers used the same methodology [33–35]. When optimizing the priors after the pilot inclusion phase, all attribute levels were dummy-coded to perform the conditional logit. For the final analysis, the variables were effects-coded. A multinomial logit model with random effects was used to analyze the respondent’s preference (Supplementary Material 2 – Eq. 1). Preference weights are reflected by the model’s beta coefficients for all levels: a significant beta coefficient for an attribute level indicates a significant impact on decision-making, the preferred attribute levels have positive coefficients and the disfavored attribute levels have negative coefficients [33]. Variance in preference for a specific attribute level between the respondents is reflected by the standard deviation (SD). A significant SD indicates significant heterogeneity between the participants in preference for an attribute. For the final analysis, the model was run using 100 draws in simulating the data and included all attributes as random effects at first instance. After this run, the attributes with non-significant SDs were moved to the fixed effects. This was the case for Daily Functioning and Gastrointestinal Complaints in the late-stage setting (Table 5). The results of running the model using 14,000 draws in data simulation are reported as final coefficients. The final utility equations can be found as Eq. 2 and 3 (Supplementary Material 2).

### Relative importance score

The attribute utility was calculated for all attributes, by calculating the absolute range between the reference level coefficient and the highest-level coefficient. For each attribute, the attribute utility was divided by the sum of all attribute utilities to derive the relative importance score per attribute.

### Trade-off assessment

First, the linearity assumption was tested for the STATA-generated coefficients for life expectancy. As life expectancy was identified as the most important aspect of care, minimum acceptable benefits were calculated to identify what improvement in life expectancy would be needed for patients to theoretically accept worsening in other attributes. This was done by first calculating the utility of a 1-month improvement in life expectancy (Supplementary Methods – Equation 4) and then calculating the quotient of change in attribute levels by the utility of a one-month improvement (Supplementary Methods – Equation 5).

### Software

Patients were registered and surveys were sent using Castor EDC [36]. STATA SE17 was used to perform conditional logit analysis to calculate optimized priors and to perform the multinomial logit model (package: mixlogit). RStudio 4.2.1 was used to process and clean data. Ngene was used to develop the initial and optimized design.

## Results

### Cohort description

After 109 completed surveys (45 late-stage disease and 64 early-stage disease), an interim analysis was performed to update the priors (Supplementary Tables 3 and 4). In the interim results, 90 patients (82%) stated that the survey was not or a little bit difficult to understand. Based on this, we concluded that the questionnaire was understandable to a sufficient number of participants, and we continued inclusion. In total, 428 individuals received a survey, of whom 255 (59.6%) started the questionnaire (Fig. 2).

**Table 3:** Baseline characteristics of all participants who included at least one choice task

	Early-stage disease (n = 165)	Late-stage disease (n = 62)
**Way of inclusion**		
Amsterdam UMC	47 (28.5%)	26 (41.9%)
PACAP	118 (71.5%)	36 (58.1%)
**Block version**		
1	24 (14.5%)	15 (24.2%)
2	21 (12.7%)	18 (29.0%)
3	23 (13.9%)	14 (22.6%)
4	39 (23.6%)	2 (3.2%)
5	27 (16.4%)	5 (8.1%)
6	31 (18.8%)	8 (12.9%)
**No other comorbidities**		
No	104 (63.4%)	36 (59.0%)
Yes	60 (36.6%)	25 (41.0%)
Missing	1	1
**Gender**		
Male	101 (63.1%)	30 (49.2%)
Female	59 (36.9%)	31 (50.8%)
Missing	5	1
**Marietal status**		
Married/living together	131 (79.4%)	49 (79.0%)
Relationship not living together	2 (1.2%)	3 (4.8%)
Single	17 (10.3%)	7 (11.3%)
Widow	13 (7.9%)	3 (4.8%)
Other	2 (1.2%)	0 (0%)
**Children**		
No	23 (14.2%)	15 (24.2%)
Yes, not living at home	49 (30.2%)	15 (24.2%)
Yes, living at home	90 (55.6%)	32 (51.6%)
Missing	3	
**Highest education**		
primary school	4 (2.4%)	0 (0%)
lower vocational education	30 (18.2%)	17 (27.4%)
higher vocational education	60 (36.4%)	23 (37.1%)
applied sciences or university	71 (43.0%)	22 (35.5%)
**Age**		
Median [Min, Max, IQR]	69.0 [46.0, 91.0, 15]	64.5 [30.0, 81.0, 14.75]
**Systemic therapy or radiotherapy**		
No and will not start	8 (4.8%)	3 (4.8%)
No, no decision made	17 (10.3%)	6 (9.7%)
No, but in the future	18 (10.9%)	10 (16.1%)
Yes, currently being treated	24 (14.5%)	17 (27.4%)
Yes, treatment has been finished	95 (57.6%)	25 (40.3%)
Unknown	2 (1.2%)	1 (1.6%)
Missing	1 (0.6%)	
**Surgery**		
No	2 (1.3%)	15 (25.4%)
Yes, in the past	132 (82.5%)	28 (47.5%)
Yes, in the future	12 (7.5%)	0 (0%)
No, depends on chemo	10 (6.3%)	1 (1.7%)
Unknown	4 (2.5%)	15 (25.4%)
Missing	5	3
**EQ5D Health today**		
Median (Min, Max, IQR)	75.0 (10.0, 100, 18)	73.0 (20.0, 100, 15)
**My complaints affect my daily functioning**		
No impact	51 (31.1%)	16 (25.8%)
Slight impact	69 (42.1%)	31 (50.0%)
Impact	35 (21.3%)	13 (21.0%)
Large impact	9 (5.5%)	2 (3.2%)
Missing	1	
**My complaints affect my appetite or eating pattern**		
No impact	81 (49.4%)	29 (46.8%)
Slight impact	44 (26.8%)	20 (32.3%)
Impact	27 (16.5%)	8 (12.9%)
Large impact	12 (7.3%)	5 (8.1%)
Missing	1	
**Interval diagnosis to survey (days)**		
Median (Min, Max, IQR)	31.4 (0.5, 90.4, 38.5)	25.4 (0.1, 90.7, 25.6)

One of the late-stage disease patients completed a questionnaire for the early-stage disease setting. Block 1–3 were survey versions that were distributed in the pilot inclusion phase. Block 4–6 were survey versions distributed in the second inclusion phase. Percentages may not sum to 100 due to rounding

**Table 4:** Attribute-level estimates for the mixed-effects logit model in the early-stage disease patient group

Attribute	Level		Coefficient	SE	z	P value
Daily functioning at three months after surgery	Daily functioning stays the same (reference)	Mean	1.016			
		SD	0.531			
	Slight worsening of daily functioning	Mean	0.574	0.095	6.03	< 0.001
		SD	−0.005	0.199	−0.03	0.979
	Clear worsening of daily functioning	Mean	−1.598	0.175	−9.11	< 0.001
		SD	0.780	0.138	5.64	< 0.001
Gastro-intestinal complaints at three months after surgery	Slight decrease of gastrointestinal complaints (reference)	Mean	0.378			
		SD	0.352			
	Gastrointestinal complaints stay the same	Mean	0.074	0.083	0.89	0.372
		SD	0.001	0.237	0	0.997
	Slight increase of gastro-intestinal complaints	Mean	−0.456	0.114	−4.02	< 0.001
		SD	0.566	0.137	4.14	< 0.001
Life expectancy after start treatment	6 months (reference)	Mean	−4.635			
		SD	1.982			
	12 months	Mean	−0.720	0.130	−5.55	< 0.001
		SD	−0.169	0.363	−0.47	0.642
	18 months	Mean	1.646	0.227	7.24	< 0.001
		SD	1.238	0.249	4.98	< 0.001
	24 months	Mean	3.673	0.406	9.04	< 0.001
		SD	2.182	0.347	6.28	< 0.001
Adverse events that cause hospitalization	0% of the patients get adverse events (ref)	Mean	0.758			
		SD	0.395			
	30% of the patients get adverse events	Mean	0.286	0.088	3.23	0.001
		SD	0.018	0.175	0.1	0.918
	60% of the patients get adverse events	Mean	−1.074	0.146	−7.34	< 0.001
		SD	0.632	0.133	4.74	< 0.001
Number of hospital visits	10 days hospital stay (ref)	Mean	0.100			
		SD	0.444			
	10 days hospital stay +6 biweekly visits	Mean	0.014	0.083	0.17	0.867
		SD	0.147	0.256	0.58	0.565
	10 days hospital stay +12 biweekly visits	Mean	−0.137	0.109	−1.26	0.207
		SD	0.685	0.134	5.09	< 0.001
Best supportive care		Mean	−15.298	3.190	−4.8	< 0.001
		SD	11.974	2.786	4.3	< 0.001

Negative coefficients for the mean indicate a negative preference for the attribute level, while positive coefficients indicate a positive preference. If not significant, the level had no effect on decision-making. A significant SD indicates significant heterogeneity regarding preferences between patients. The sign of the SD is not relevant and can be interpret as positive. SE = standard error, SD = standard deviation

There were 28 participants who only completed the questions regarding demographics and clinical history. 227 participants answered at least one choice task and were included in the analysis. In the early-stage disease setting, participants who did not start any choice task went to primary school or lower vocational education more often (12/20 (60.0%) vs 34/162 (21.0%), overall *p* = 0.008). Patients with late-stage disease who did not start the choice tasks more often did not receive chemotherapy or were still planning to start (6/9 (66.7%) vs 13/62 (21.0%)), while of the choice task-responders, more participants were currently receiving chemotherapy or underwent therapy in the past (2/9 (22.2%) vs 42/62 (67.7%), overall *p* = 0.02). Participant characteristics are shown in Table 3. One patient accidently received a survey for the early-stage disease instead of late-stage disease, resulting in 165 responses for early-stage disease patients and 62 responses for late-stage disease patients.

### Patients-reported preferences for patients with early-stage disease

**Fig. 3:**
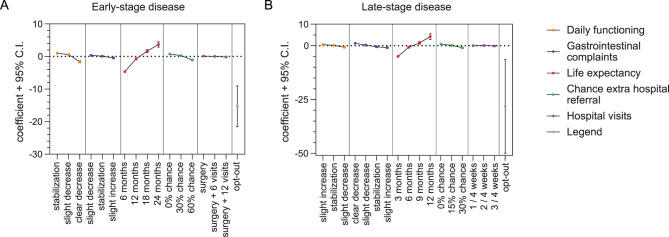
Attribute-level estimates for the mixed effects logit model. Mixed logit coefficients and 95% confidence intervals (C.I.) are presented for each level, except for the reference level. Negative coefficients indicate a negative effect of this level when making a treatment decision. Positive coefficients indicate a positive preference for a certain attribute level. When the 95% C.I. comprises zero, the attribute level had no significant effect on decision-making. The opt-out option was an equivalent of best supportive care. (**A**) coefficients for the early-stage disease setting patient group. (**B**) coefficients for the late-stage disease setting patient group

The results of the mixed logit assessment for preferences in the early-stage disease patient group are presented in Table 4 and Fig. 3A. All attributes included in the model, except for the number of hospital visits, were found to be relevant for decision making for at least one level. If the treatment would cause a stabilization of gastrointestinal complaints, instead of a decrease, decision-making was not significantly affected. There was a significant negative coefficient for receiving BSC, indicating the participants’ preference to receive an anti-tumor therapy instead of BSC only. All attributes had a significant SD for at least one level, meaning that within the participants, significant heterogeneity existed regarding the importance of these attributes in decision-making. This heterogeneity appeared particularly in the higher attribute levels, such as a greater decrease in daily functioning, a larger increase in gastrointestinal complaints, a higher risk of adverse events and more hospital visits. The significant SD for BSC indicates that the participants had heterogeneous preferences towards BSC as well.

**Table 5:** Attribute-level estimates for the mixed-effects logit model in the late-stage disease patient group

Attribute	Level		Coefficient	SE	z	P value
Daily functioning three months after start chemotherapy	Slight increase in daily functioning (reference)	Mean	0.505			
	Daily functioning stays the same	Mean	0.189	0.152	1.240	0.216
	Slight decrease of daily functioning	Mean	-0.694	0.172	-4.030	<0.001
Gastro-intestinal complaints three months after start chemotherapy	Clear decrease in gastro-intestinal complaints (reference)	Mean	1.115			
	Slight decrease in gastro-intestinal complaints	Mean	0.272	0.197	1.380	0.168
	Gastrointestinal complaints stay the same	Mean	-0.506	0.190	-2.660	0.008
	Slight increase in gastro-intestinal complaints	Mean	-0.881	0.194	-4.530	<0.001
Life expectancy after start treatment	3 months (reference)	Mean	-4.975			
		SD	1.806			
	6 months	Mean	-0.677	0.224	-3.020	0.003
		SD	-0.225	0.496	-0.450	0.650
	9 months	Mean	1.304	0.301	4.330	<0.001
		SD	-0.603	0.387	-1.560	0.119
	12 months	Mean	4.251	0.673	6.320	<0.001
		SD	2.420	0.571	4.240	<0.001
Advere events that cause hospitalization	0% of the patients get adverse events (ref)	Mean	0.725			
		SD	0.700			
	15% of the patients get adverse events	Mean	0.169	0.164	1.030	0.302
		SD	0.342	0.383	0.890	0.372
	30% of the patients get adverse events	Mean	-0.922	0.229	-4.020	<0.001
		SD	1.056	0.235	4.490	<0.001
Number of hospital visits	1 visit per 4 weeks (ref)	Mean	0.029			
		SD	0.344			
	2 visits per 4 weeks	Mean	0.138	0.144	0.960	0.339
		SD	0.044	0.311	0.140	0.886
	3 visits per 4 weeks	Mean	-0.162	0.165	-0.980	0.327
		SD	-0.595	0.218	-2.740	0.006
Best supportive care		Mean	-28.203	11.109	-2.540	0.011
		SD	17.936	7.016	2.560	0.011

Negative coefficients for the mean indicate a negative preference for the attribute level, while positive coefficients indicate a positive preference. If not significant, the level had no effect on decision-making. A significant SD indicates significant heterogeneity regarding preferences between patients. The sign of the SD is not relevant and can be interpret as positive. Std.err. = standard error, SD = standard deviation

### Patient-reported preferences for patients with late-stage disease

Results for the late-stage disease patient group are shown in Table 5 and Fig. 3B. Like the patients with early-stage disease, the number of hospital visits was not relevant in decision-making and the participants preferred to receive anti-tumor treatment instead of only BSC. If a treatment option caused a worsening of daily functioning or gastrointestinal complaints, or a 30% risk of adverse events, this had a significant negative effect on the selection of this treatment. The SDs for daily functioning and gastrointestinal complaints were not significant, indicating that these attributes were of similar relevance to all participants. Similar to the early-stage disease setting, SDs were statistically significant for the higher levels of life expectancy, risk of adverse events and number of hospital visits, as well as for receiving BSC instead of anti-tumor treatment. Consequently, there was significant heterogeneity between the patients in their preference for BSC instead of anti-cancer treatment.

### Life expectancy vs quality of life trade-off

**Fig. 4:**
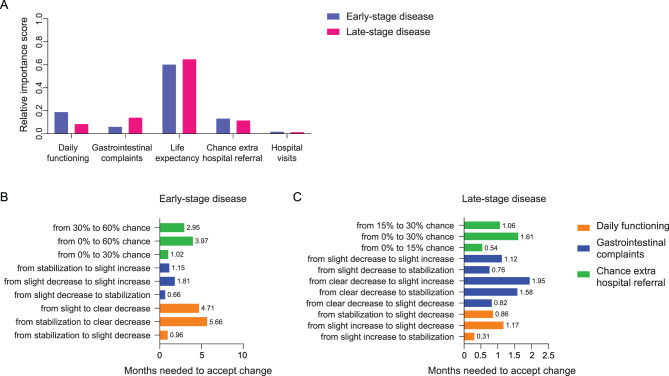
Level-specific utility scores. (**A**) relative importance scores per attribute and treatment setting. Scores for all attributes in one setting sum to one. (**B**) months of increased life expectancy needed to accept a certain level of side effects in the early-stage disease setting. (**C**) months of increased life expectancy needed to accept a certain level of side effects in the late-stage disease setting. In both settings, hospital visits were not relevant in decision-making and therefore not included. Horizontal bars depict how many months of life expectancy should be added to compensate for the utility loss caused by a change in side effects or quality of life

First, the relative importance scores were calculated per attribute (Fig. 4A). In both questionnaire settings, life expectancy had the highest relative importance score and was the most important attribute in decision-making. In the early-stage disease setting, life expectancy was regarded a factor 3.2 more important by patients than daily functioning, which was the second most important attribute. In the late-stage disease setting, life expectancy was regarded a factor 4.6 more important than gastrointestinal complaints, the second most important attribute in this setting. Next, we calculated how many months a treatment should prolong a patient’s life expectancy to accept unwarranted effects of treatment (Fig. 4B, C). To accept a treatment for early-stage disease causing a clear decrease in daily functioning, a slight increase in gastrointestinal complaints and a 60% chance of adverse events, this treatment should cause an increase in life expectancy of 11.5 months. In late-stage disease, a treatment causing a slight increase in gastrointestinal complaints, a slight decrease in daily functioning and 30% chance of adverse events, was deemed acceptable if it prolonged life expectancy with 4.7 months.

### Subgroup analyses

Above, the results for all participants who completed at least one choice task were presented. For the PACAP-included patients, more detailed clinical information became available after completing the survey and this was used to perform subgroup analyses. In the early-stage disease cohort, one patient did not undergo surgery because of comorbidities and six patients had recurrent disease in their follow-up. Repeating the analysis while excluding these patients, including the late-stage disease patient who received an early-stage disease survey, led to similar results (Supplementary Fig. 2, Supplementary Table 5).

Patients were selected for the late-stage disease treatment cohort based on the absence of a curative treatment plan in the NCR. However, 15 patients in this setting received systemic treatment for a locally advanced pancreatic cancer but underwent surgery afterwards. These patients had a longer interval between diagnosis and survey inclusion than the other patients in the palliative cohort (median interval = 37.7 months vs. 21.0 months, *p* = 0.001). These patients were excluded in a sensitivity analysis, and it was assessed if the remaining patients still had a significant negative preference towards the BSC option. Executing the first modeling step resulted in a model with significant SDs for each attribute. When the number of draws was increased to increase accuracy of the model, there was a significant negative preference towards BSC, but also a lot of variation between patients (coefficient = −491 & *p* < 0.001, SD = 456 & *p* < 0.001, Supplementary Table 6). When Daily Functioning and Gastrointestinal Complaints were treated as fixed effects like the model in the complete late-stage disease cohort, the model estimates were similar to the original complete cohort model (Supplementary Fig. 3). The preference for BSC was significantly negative again although the heterogeneity between patients was not significant (coefficient = −28.0 & *p* = 0.037, SD = 22.0 & *p* = 0.053, Supplementary Table 7).

### Patient-reported importance of attributes

**Fig. 5:**
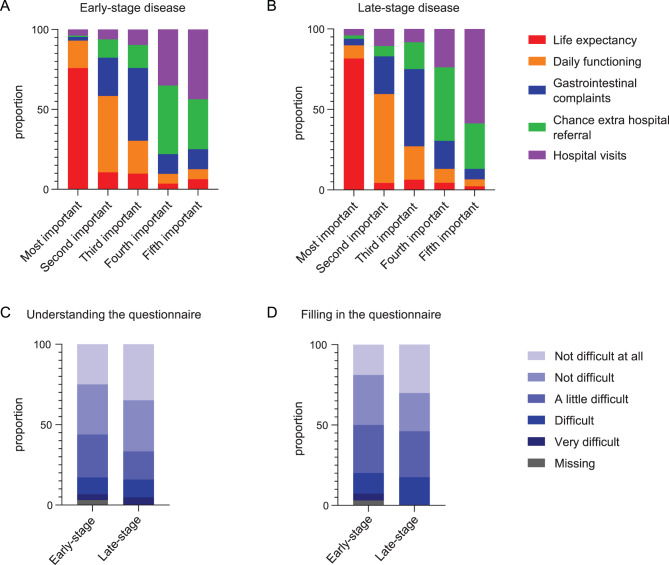
Participant feedback to the survey. Ranking of attributes by the early-stage (**A**) and late-stage (**B**) disease patient group from most to least important. When multiple criteria were ranked at the same level of importance, they were scored as missing. (**C**) feedback to the question “was it hard to understand the questionnaire?”. (**D**) feedback to the question: “was it hard to fill in the questionnaire?”

At the end of the questionnaire, participants were asked to rank the included attributes from most to least important. Some participants did not follow the intended instructions correctly. For instance, some marked one attribute as the most important, while designating the remaining four attributes as equally important. Therefore, only levels of importance at which one attribute was mentioned, were included in the analysis and the others were scored as missing (22–32% of the responses in the early-stage disease setting and 21–26% of the responses in the late-stage disease setting were missing; Fig. 5A, B). The same pattern was observed for both settings, with ‘life expectancy’ as most important attribute, ‘daily functioning’ as second important attribute, ‘gastrointestinal complaints’ as third important attribute, ‘chance for an extra hospital referral’ as fourth important attribute and ‘number of hospital visits’ as fifth important attribute. Some patients stated that they missed attributes of the treatment or criteria that are important to patients in their decision-making such as pain, mental status of the patient, relationship with physician, the duration of the visits rather than the number of the visits, and a chance of cure.

### Feedback to the questionnaire

The responses in the feedback section of the questionnaire highlighted some misconceptions between the designers of the study and the participants. For example, some participants missed general quality of life as an attribute, which we intended to be covered by ‘daily functioning’. Another participant stated that nausea was missing as an attribute, which we intended to be covered under ‘gastrointestinal complaints’. However, the majority of the participants who did complete at least one choice task, considered the survey as not or only a little difficult to understand (Fig. 5C). Completing the survey was more difficult to the participants than understanding the survey (Fig. 5D). 41.2% of the early-stage disease group (68/165) and 32.3% of the late-stage disease group (20/62) thought that it was a little difficult, difficult, or very difficult to understand the survey, while for completing the survey, this was 47.3% for the early-stage disease group (78/165) and 45.2% for the late-stage disease group (28/62).

## Discussion

Here we present the first multicenter discrete-choice experiment and one of the largest studies into treatment preferences for patients with pancreatic cancer, including patients with early- and late-stage disease. Our main finding is that both patient groups showed a strong preference to receiving treatment instead of best supportive care. Importantly, the standard deviations for the BSC coefficient were also significant, indicating that this strong preference for treatment was not consistent across respondents. The same was observed in the worse-perceived levels of other attributes, such as a higher risk of adverse events. This suggests that while some patients had a strong preference to avoid these unfavourable outcomes, others were less concerned. This heterogeneity stresses the importance of shared decision-making efforts in pancreatic cancer care. Shared decision-making improves patients’ satisfaction, quality of life, and acceptance of palliative care [37, 38]. In the Netherlands, an online shared decision-making tool was designed for patients with metastatic disease to support personalized decision-making based on individual preferences [39]. This will likely enable physicians to identify which patients prioritize life expectancy and who prioritizes a balance between life expectancy and quality of life.

To answer our research question, a mixed-method approach was adopted, starting with a literature study and interviews during the design phase to identify relevant treatment attributes. However, qualitative approaches do not allow for quantification of patients’ preferences or estimation of trade-offs. A discrete choice experiment (DCE), on the other hand, is specifically designed to provide such quantitative estimates and has become an established and widely used method in health economics and preference research.[40–44]

Preference patterns in early-stage and late-stage disease patients were similar, with the greatest emphasis placed on life expectancy. The importance of life expectancy in patients with late-stage disease was in line with the study performed by Pihlak et al., who observed that patients with irresectable pancreatic cancer often prioritise the longest possible survival over a balance between side-effects and survival [45]. In line with these results, the BSC options had strong negative coefficients in both settings, even though some of the choice tasks provided an imaginary life expectancy of only one or two months longer than BSC. This indicates that patients have a strong preference towards receiving anti-tumour treatment instead of best supportive care, even when the expected benefits are low. Similar results have been observed before in other subgroups of cancer patients [46–48]. In contrast, a large study into several metastatic cancer types showed that the majority of the patients thought that length and quality of life were equally important.[49]

The strong preference for anticancer treatment as shown in our study might reflect population-wide preferences or may stem from specific characteristics of our study cohort. In general, cancer treatment decisions are not only based on the risks and benefits of a certain treatment, but also on patients’ and physicians’ attitudes and on how the information is presented [50]. For example, in advanced cancer patients, the term ‘palliative care’ was stigmatized and reduced to end-of-life comfort care [51]. Patients eligible for phase 1 trials did not consider palliative treatment as a considerable option for themselves and almost all of them would participate in a trial with a high risk of serious adverse effects [52]. In the curative setting, a study interviewing early-stage breast cancer patients who had to decide about adjuvant treatment illustrated that refraining from adjuvant treatment was not a realistic option to them [53]. Actively deciding for treatment gave them a sense of control over their disease. Additionally, hope to get cured still might play a role in advanced cancer care. Therapeutic misconception or misestimation and unrealistic optimism regarding prognosis were reported amongst advanced cancer patients in phase 1 trials [54] and at regular outpatient clinics [55], probably caused by unrealistic and dispositional optimism regarding therapeutic benefit [56]. Finally, pancreatic cancer has a notorious reputation because of its deadliness [57] and this might contribute to a higher acceptance of a small gain in life expectancy regardless of the side effects.

However, despite of the abovementioned mechanisms and the strong reported preference towards anti-cancer treatment in this study, the majority of the Dutch pancreatic cancer patients does not get anticancer treatment [6, 7]. This is most likely explained by a combination of patients’ preferences and the disease-related frailty of pancreatic cancer, rather than by physicians’ non-adherence to best practices alone [7, 58, 59]. Therefore, the reported patients’ preferences in this particular study might be affected by the respondents’ characteristics. Importantly, most of the respondents were eligible for treatment or underwent a successful treatment in the past, as indicated by their active participation in the PACAP questionnaires and their relatively long survival. These characteristics and experiences could have affected their choices in the survey. Having previously made a treatment decision could lead to bias when reflecting on that decision or when faced with a similar decision. This bias may stem from a positive experience with the previous treatment or from cognitive dissonance reduction—a process in which patients seek consistency between past and current behaviour leading them to favour the same treatment [47, 60–62]. In contrast, patients with negative treatment experiences were probably less likely to start this survey. Furthermore, patients receiving BSC only were hard to reach because of their short life expectancy, poor condition and less regular hospital visitation, and were therefore under-represented in this study. Due to these mechanisms, participants in this survey are expected to be biased towards preferring treatment options over BSC. Despite of the biased population of our cohort, the preferences of our participants are relevant to this research aim because of their treatment experience. By undergoing treatment, they experienced the side effects of chemotherapy and the recovery of pancreatic surgery. They could execute the fictional choice tasks based on this experience.

Compared to other studies, our study cohort was representative for pancreatic cancer patients regarding age and EQ5D health status as reported by others [6, 63–65]. However, the median time interval from date of diagnosis to completion of the survey was particularly high for the late-stage disease patients: 25.1 months, indicating a long survival since diagnosis. To explain this, we speculate that long treatment responders are over-represented in the study as an effect of survivorship bias. After all, almost a fifth of metastatic patients can experience a long treatment response that exceeds eighteen months or more [17]. Besides, some patients had metachronous metastases. However, as mentioned before, 15 patients were registered in the NCR as non-curative for having locally advanced disease but underwent surgery. But excluding these patients still showed a significant negative preference towards BSC. Unfortunately, it was not possible to recruit a larger number of patients in the palliative setting. In a simulation DCE, it was shown that precision of the study rapidly increases per extra included respondent until a sample size of 150 [32], a number that was not reached for cohort of late-stage disease patients. Consequently, the calculated coefficients for this setting should be interpreted with caution and the results of the late-stage disease treatment surveys should be considered as hypothesis-generating rather than conclusive.

Even though the attributes and levels were designed to reflect treatment guidelines for pancreatic cancer, not all treatment settings were represented. Around 30% of the patients with pancreatic cancer has borderline resectable or locally advanced disease [66]. These patients might benefit from neo-adjuvant chemotherapy or chemoradiation which might allow for radical resection after restaging [67]. These treatments introduce extra uncertainty in the treatment process when added to resection and adjuvant treatment. Incorporating them in a DCE would necessitate the addition of an extra treatment attribute, ‘chance for tumour resection’, and an extra cohort of solely neo-adjuvant treated patients. Because of feasibility aspects, we included neo-adjuvant treated patients in the survey according to the most likely treatment setting, which was often the early disease stage setting. Future research should assess how patients weigh the risks of a higher chance of a resection against the side effects of neoadjuvant treatment. Another limitation of the study is that the survey questions might be too demanding to understand and complete for a proportion of the patients. This might harm the generability of the results. However, the fact that some participants did not follow the instructions at the ranking exercise at the end of the survey, does not imply that they failed to understand the main choice tasks as well. DCEs have been widely and successfully applied in other cancer types [40–43], demonstrating that they are an accepted and appropriate methodology for exploring patient preferences in early economic evaluations. Lastly, although participants could provide feedback on the survey during both the design and including phase of the study, we did not formally measure the emotional state of the patients before and after completing the survey. Despite the widely accepted use of DCEs in cancer research and our clear instructions to participants that the survey was hypothetical and not related to their personal situation, future studies may benefit from formally assessing the emotional impact of the survey during the design and pilot phases, to ensure that any burden is minimized.

## Conclusions

This is the first DCE conducted in pancreatic cancer, thereby providing novel data on how patients weigh survival and quality-of-life trade-offs. Even when no curative options are provided, a substantial proportion of patients prefer to receive life-extending treatment over best supportive care. However, the substantial variability in responses underscores the importance of individualized, transparent communication about prognosis, treatment goals, and patient wishes, we believe our findings reinforce and make more tangible the relevance of patient-centered care in this disease.

## Electronic supplementary material

Below is the link to the electronic supplementary material.

Supplementary Material 1

Supplementary Material 2

Supplementary Material 3

Supplementary Material 4

Supplementary Material 5

Supplementary Material 6

## Data Availability

The data underlying this article cannot be shared publicly due to the privacy of the respondents. The data will be shared on reasonable request to the corresponding author. The data dictionary and used scripts can be requested from the corresponding author.
