# Supplementary material for: Patient preferences for pancreatic cancer treatment (PERSEUS): a multicenter discrete choice experiment
Source: Health Qual Life Outcomes. 2025 Dec 24;23:122. doi: 10.1186/s12955-025-02440-5 (PMC12729412; doi:10.1186/s12955-025-02440-5)
Supplement: Supplementary file 1 — Supplementary Material 1 [file 12955_2025_2440_MOESM1_ESM.pdf]

General introduction

First, thank you very much for your participation in this research. In this research, we aim to study what is of importance to patients regarding their pancreatic cancer treatment.

To do this, we use a survey consisting of three parts. In part A, we ask you some questions about your background. In part B, we first provide you with some information about **imaginary treatments,** that are not related to your own situation. After this, we ask you to decide several times between two imaginary treatment options. For each decision, you should keep the following question in the back of your mind: “Which one of these two treatments would I prefer?”. In part C, you can give feedback about the survey.

In part B we give quite a lot of information. Some of the questions can be complicated. That’s why we ask you to take your time to complete this survey.

Thank you again very much for your participation.

With kind regards,

Part A: questions about your background

First, we ask you some general questions.

1. What is your age? ……….

2. Do you have pancreatic cancer according to your medical doctors?
   - O Yes
   - O No

3. Do you have any other diseases / conditions at this moment, according to your doctors? If yes, can you specify these? You can give multiple answers.
   - O I don't have other diseases or conditions
   - O Pancreatitis (inflammation of the pancreas)
   - O Cancer of the bile ducts, duodenum or ampulla
   - O Neuroendocrine tumor (NET)
   - O Cysts, like a IPMN
   - O Another type of cancer (at this moment or in the past), which is: ………………………………………………………………………………….
   - O Arthritis (inflammation of the joints)
   - O Asthma
   - O Anaemia
   - O COPD
   - O Dementia or mild cognitive impairment
   - O Depression
   - O Diabetes
   - O Heart failure or other cardiovascular diseases
   - O Hypertension (high blood pressure)
   - O Liver diseases
   - O Kidney diseases or kidney failure
   - O Obesity (severe overweight)
   - O Other: …………………………………………………………………………

The next question (question 4) should only be answered if you have indicated to have other conditions than pancreatic cancer:

4. Do the symptoms or the treatments of these conditions affect your choice of treatment for pancreatic cancer
   - O Yes
   - O No
   - O I don't know

5. What is your sex?
   - O Male
   - O Female
   - O I prefer not to say
   - O Other: …………………………………………………………………………

6. What is your current marietal status?
   - O Married / living together
   - O In a relationship, not living together
   - O Single
   - O Widowed / partner has deceased
   - O Other:……………………………………………………………………………………………….

7. Do you have children?
   - O Yes, they live at home
   - O Yes, they don't live at home
   - O No

8. What is your highest finished level of education?
   - O Primary school
   - O Pre-vocational education
   - O Secondary vocational education
   - O Higher professional education, university of applied sciences, university

9. In which hospital are you undergoing / did you undergo your treatment? You can give multiple answers.
   - O Amphia hospital
   - O Amsterdam UMC (both locations)
   - O Sint Antonius hospital
   - O Catharina hospital
   - O Erasmus MC
   - O Isala
   - O Jeroen Bosch hospital
   - O Leiden UMC
   - O Maasstad hospital
   - O Maastricht UMC
   - O Medical Spectrum Twente
   - O OLVG
   - O Radboud UMC
   - O UMC Groningen
   - O UMC Utrecht
   - O Other:………

10. Have you started a treatment with chemotherapy or radiotherapy (irradiation)?
    - O No, and I **will not start** a treatment with chemotherapy or radiotherapy.
    - O No, and I **have not made a decision yet** about my treatment.
    - O No, but I **am going to start** a treatment (soon).
    - O Yes, I am undergoing treatment at this moment.
    - O Yes, I have finished my treatment.

11. Did you undergo or will you undergo pancreatic surgery?
    - O Yes, I underwent pancreatic surgery **in the past**.
    - O Yes, I will undergo pancreatic surgery **in the future**.
    - O Yes, but **only** if the chemotherapy or radiotherapy is successful.
    - O No.
    - O I don't know / unknown.

12. Can you indicate which sentence fits best to your health situation **today:**
    Do you experience problems with: Walking
    - O I don't have problems with walking.
    - O I have some problems with walking.
    - O I have moderate problems with walking.
    - O I have severe problems with walking.
    - O I am not able to walk.

13. Can you indicate which sentence fits best to your health situation **today:**
    Do you experience problems with: Personal hygiene
    - O I don't have problems with washing myself or getting dressed.
    - O I have some problems with washing myself or getting dressed.
    - O I have moderate problems with washing myself or getting dressed.
    - O I have severe problems with washing myself or getting dressed.
    - O I am not able to wash myself or getting dressed.

14. Can you indicate which sentence fits best to your health situation **today:**
    Do you experience problems with: Performing daily activities (work, housekeeping, family, education, hobbies)
    - O I don't have problems with performing daily activities.
    - O I have some problems with performing daily activities.
    - O I have moderate problems with performing daily activities.
    - O I have severe problems with performing daily activities.
    - O I am not able to perform daily activities

15. Can you indicate which sentence fits best to your health situation **today:**
    Do you experience: Pain / discomfort
    - O I don't have pain / discomfort.
    - O I have some pain / discomfort.
    - O I have moderate pain / discomfort.
    - O I have severe pain / discomfort.
    - O I have extreme pain / discomfort.

16. Can you indicate which sentence fits best to your health situation **today:**
    How is your mood?
    - O I am not scared or feeling sad.
    - O I am a bit scared or feeling a bit sad.
    - O I am moderately scared or feeling moderately sad.
    - O I am very scared or feeling very sad.
    - O I am extremely scared or feeling extremely sad.

17. How is your health situation **today**?

*This scale goes from 0 to 100. 100 indicates the <u>best</u> health you can imagine. 0 indicates the <u>worst</u> health you can image. Please indicate your health situation **today** on the scale.*

the worst health you can imagine

the best health you can imagine

0 10 20 30 40 50 60 70 80 90 100

At this moment, you might experience complaints caused by the disease or the treatment. Could you indicate in what extent these complaints have an effect on:

18. My daily activities:
    - O No impact on my daily activities
    - O A little bit impact on my daily activities
    - O Impact on my daily activities
    - O A lot of impact on my daily activities

19. My appetite and eating pattern:
    - O No impact on my appetite and eating pattern
    - O A little bit impact on my appetite and eating pattern
    - O Impact on my appetite and eating pattern
    - O A lot of impact on my appetite and eating pattern

Part B: Which treatment has your preference?

In this part, we ask you to choose a couple of times between two **imaginary treatments.**

Treatments can have all kind of **characteristics**. For example, the side effects. The characteristics are described in levels: for example, one chemotherapy has severe side effects while another chemotherapy causes mild side effects. For this survey, we chose to describe the treatment options using the same five characteristics every time. We will explain these characteristics step-by=step to you.

These are the characteristics we use in the survey:

**Number of hospital visits**	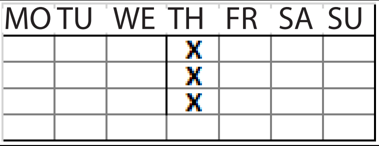
**Chance for an extra hospital referral**	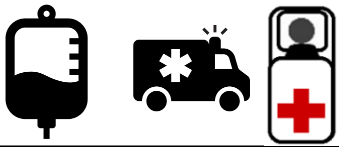
**Daily functioning after three months**	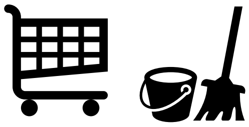
**Gastrointestinal (abdominal) complaints after three months**	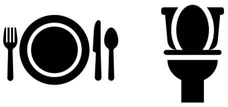
**Life expectancy**	

Number of hospital visits

By *number of hospital visits,* we mean how many visits to the hospital are needed to undergo the treatment against cancer. These are treatments that let the tumor shrink or remove the tumor. You can think of staying in the hospital after surgery or visits to receive chemotherapy. These visits are scheduled in advance. Visits aiming to relieve complaints, such as pain relief, are not taken into account.

In the survey, we use the number of visits per months. We show a calendar and the crosses indicate which days you should be in the hospital. The calendar below indicates that in this months, you should visits the hospital at three days:

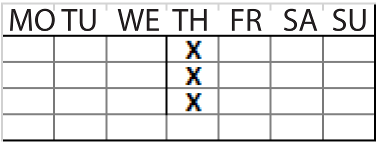

In this survey, the *number of hospital visits* is described like this:

Tekst in vragenlijst	Uitleg
1 visit per 4 weeks 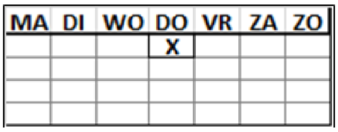	One visit each four weeks to receive chemotherapy
2 visits per 4 weeks 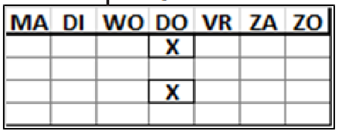	Two visits each four weeks (one visit every two weeks) to receive chemotherapy
3 visits per 4 weeks 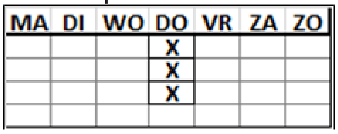	Three visits each four weeks (one visit every week for three weeks and then a week no visit) to receive chemotherapy

Chance for an extra hospital referral

Some treatments can cause complaints, like side effects of chemotherapy. Sometimes, these complaints are severe and an *extra hospital referral* will be needed. During such a referral, it might for example be necessary to do a blood transfusion, receive antibiotics or perform an extra, unscheduled, surgery. Some patients will need an extra referral to the hospital, but it is uncertain if this will happen to you as well.

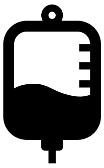 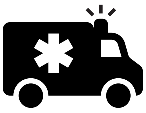

In the survey we show which part of all patients who are getting the treatment, will need an extra hospital referral. We do it like this: we show a group of hundred patients. Not all patients get severe complaints. The patients who do no get severe complaints, are displayed like:

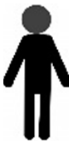

The patients who do get severe complaints and need an extra hospital referral, are displayed like:

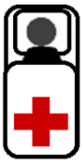

In the survey we describe the number of patients that need an extra hospital referral as followed:

text in survey	explanation
0 out of 100 patients (0%) 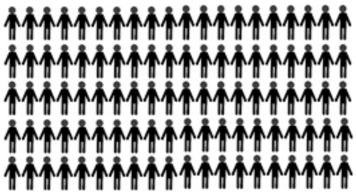	None of the 100 patients (0%) gets serious complaints caused by the treatment and nobody needs an extra hospital referral
15 out of 100 patients (15%) 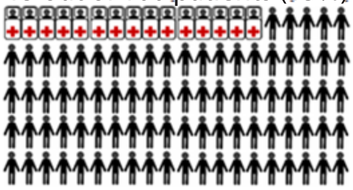	15 of the 100 patients (15%) gets serious complaints caused by the treatment and nobody needs an extra hospital referral
30 out of 100 patients (30%) 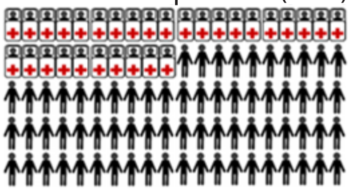	30 of the 100 patients (30%) gets serious complaints caused by the treatment and nobody needs an extra hospital referral

Question to practice

In this survey we combine characteristics to described imaginary treatments. For the two characteristics that we have discussed already, the description could look like this:

Treatment 1	
Number of hospital visits	2 visits per 4 weeks 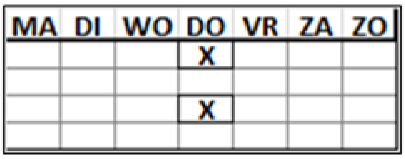
Chance for an extra hospital referral	15 out of 100 patients (15%) 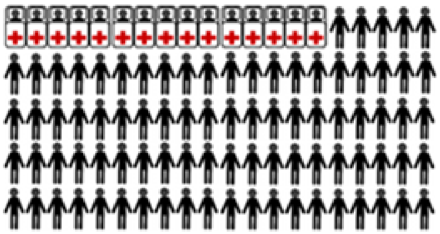

Imagine that a patient would choose Treatment 1, are these sentences **true** or **false**?

1. **30%** of the patients will get severe complaints and need to be referred to the hospital.
   - O true
   - O false
2. The patient has to visit the hospital **one time** per month to receive the treatment.
   - O true
   - O false

The answers can be found on the next page.

Sentence 1 was **false.** In this imaginary treatment, 15 of the 100 patients (15%) will need an extra hospital referral because of severe complaints. This is less than 30% of the patients (30 of the 100).

15 of 100 patients (15%)

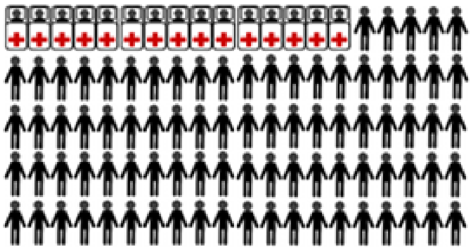

is less than

30 of 100 patients (30%)

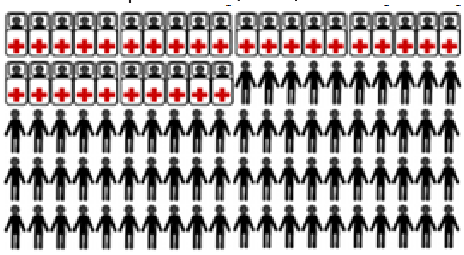

Sentence 2 was **false**. To get this imaginary treatment, patients have to visits the hospital two times per month to receive their treatment.

Daily functioning at three months after the start of the first cycle of chemotherapy

By *daily functioning* we mean: everything you would do on a normal day, like going to work or housekeeping. As an effect of the treatment, you might be able to do more or less activities on a days, for example because you are feeling tired.

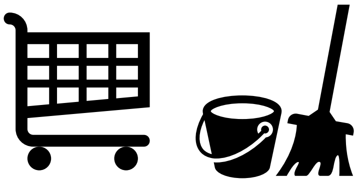

As a result of chemotherapy, your *daily functioning may deteriorate*. You may be able to do less in a day than before chemotherapy began. It may also happen that your *daily functioning improves*. You may be able to do more in a day than before.

In this survey we describe how daily functioning will have changed **three months after the first treatment** compared to **just before the first treatment**, like this:

text in survey	explanation
Slightly noticable worsening 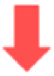	Daily functioning has slightly noticable worsened, compared to before the first cycle of chemotherapy.
No change 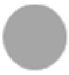	Daily functioning has stayed the same, compared to before the first cycle of chemotherapy.
Slightly noticable increase 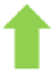	Daily functioning has slightly noticable increased, compared to before the first cycle of chemotherapy.

Abdominal complaints three months after the first treatment

You can experience *abdominal complaints*, like a reduced appetite, nausea, vomiting, constipation or diarrhea. You could get more or less abdominal complaints than before the start of the treatment. You could also experience other abdominal complaints because of the treatment.

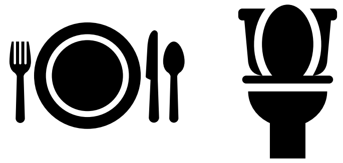

It can happen that the chemotherapy causes the *abdominal complaints to increase*. For example, you will have diarrhea more often. But it can also happen that the *abdominal complaints decrease*. For example, you will have less abdominal pain and more appetite than before you started treatment.

In this survey we describe how the abdominal complaints will have changed **three months after the first course of treatment** compared to **just before the first course of treatment**, like this:

text in survey	explanation
Slightly noticable worsening	Gastrointestinal complaints are slightly noticable worsened compared to before the first cycle of chemotherapy
No change	Gastrointestinal complaints are the same as compared to before the first cycle of chemotherapy
Slightly noticable decrease	Gastrointestinal complaints are slightly noticable decreased compared to before the first cycle of chemotherapy
Clearly noticable decrease	Gastrointestinal complaints are clearly noticable decreased compared to before the first cycle of chemotherapy

Life expectancy

Life expectancy refers to: the expected number of months you will live from the first chemotherapy. This questionnaire is about an **imaginary** situation. This is therefore not your situation.

In this survey, we describe life expectancy like this:

text in survey	explanation
3 months	People who choose this intervention, live until three months after treatment start
6 months	People who choose this intervention, live until six months after treatment start
9 months	People who choose this intervention, live until nine months after treatment start
12 months	People who choose this intervention, live until twelve months after treatment start

Question to practice

In this survey we combine all the properties to describe imaginary treatments. For the three properties we just discussed, it could look like this:

Treatment 1	
Daily functioning	Slightly noticable increase<br>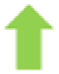
Abdominal complaints	Clearly noticable decrease<br>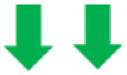
Life expectancy	12 months

Suppose a patient were to choose Treatment 1, what would be the consequences for this patient?

1. The patient will be able to do less / more housework than before starting treatment.
2. The patient will suffer less often / more often from nausea.
3. The patient will likely live less / longer than 10 months after the first chemotherapy treatment.

The answers can be found at the bottom of the page:

The answer to Question 1 was: **more**. Because the patient has a better daily functioning, the patient can do more things in a day. The patient can also do more in the housework.

The answer to Question 2 was: **less often**. Because the patient's abdominal complaints have decreased, the patient is less often nauseous. Nausea is part of the abdominal complaints.

The answer to Question 3 was: **longer.** The patient’s life expectancy is 12 months. This is more than 10 months.

Part B: which treatment has your preference?

We now ask you to choose between imaginary treatment A and B. You can keep the following question in mind: "If I had to choose between these two treatments, which would I prefer?". What you choose in the questionnaire does not determine which treatment you will receive in reality. If neither treatment appeals to you, you can also choose to receive only **basic care**.

Basic care is the care that all patients receive to relieve their symptoms. Basic care will make you feel a little better and have fewer symptoms. An example of basic care is giving painkillers. You rarely have to go to the hospital for basic care. If you only choose basic care, you will not receive treatment that tries to shrink or remove the tumor. Of the patients who only receive basic care, one half will live for less than two months and the other half will live for more than two months.

If you want to provide feedback about the survey, you can do so at the end of the survey in Part C.

Choice task 1

Could you indicate if you would choose for **Treatment A, Treatment B** or **Basic care:**

	**Treatment A**	**Treatment B**	**Basic care**
Number of hospital visits	3 visits per 4 weeks<br>MA DI WO DO VR ZA ZO<br>DO: X X X	1 visit per 4 weeks<br>MA DI WO DO VR ZA ZO<br>DO: X	- No treatment that removes the cancer or makes it smaller.<br>- Only appointments in the hospital when there are complaints.<br>- Because of the basic care, you will have less complaints and feel a bit better.<br>- Halve of the patients who receive only basic care, lives shorter than two months. The other halve lives longer.
Chance for an extra hospital referral	0 of 100 patients (0%)<br>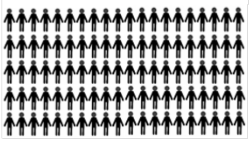	30 of 100 patients (30%)<br>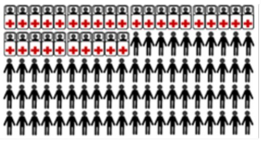	
Daily functioning after three months	No difference	Slightly noticable worsening	
Gastrointestinal complaints after three months	Slightly noticable increase	No difference	
Life expectancy	**3 months**	**9 months**	

I choose: Treatment A / Treatment B / Basic care
(circle your choice)

Choice task 2
Could you indicate if you would choose for **Treatment A, Treatment B** or **Basic care:**

	**Behandeling A**	**Behandeling B**	**Basiszorg**
Aantal ziekenhuisbezoeken	3 bezoeken per 4 weken<br>MA DI WO DO VR ZA ZO<br>X<br>X<br>X	1 bezoek per 4 weken<br>MA DI WO DO VR ZA ZO<br>X	- Geen behandeling die de kanker weghaalt of kleiner maakt.<br>- Alleen afspraken in het ziekenhuis bij klachten.<br>- Door de basiszorg heeft u minder klachten en u voelt zich wat beter.<br>- De helft van de patiënten die alleen basiszorg krijgt, leeft korter dan twee maanden. De andere helft leeft langer.
Kans op een extra ziekenhuisopname	15 van de 100 patiënten (15%)	0 van de 100 patiënten (0%)	
Dagelijks functioneren na drie maanden	Net merkbare verslechtering	Geen verschil	
Buikklachten na drie maanden	Duidelijk merkbare afname	Net merkbare toename	
Levensverwachting	3 maanden	3 maanden	

I choose: Treatment A / Treatment B / Basic care
(circle your choice)

Choice task 3
Could you indicate if you would choose for **Treatment A, Treatment B** or **Basic care:**

	**Behandeling A**	**Behandeling B**	**Basiszorg**
Aantal ziekenhuisbezoeken	3 bezoeken per 4 weken<br>MA DI WO DO VR ZA ZO<br>X<br>X<br>X	1 bezoek per 4 weken<br>MA DI WO DO VR ZA ZO<br>X	- Geen behandeling die de kanker weghaalt of kleiner maakt.<br>- Alleen afspraken in het ziekenhuis bij klachten.<br>- Door de basiszorg heeft u minder klachten en u voelt zich wat beter.<br>- De helft van de patiënten die alleen basiszorg krijgt, leeft korter dan twee maanden. De andere helft leeft langer.
Kans op een extra ziekenhuisopname	0 van de 100 patiënten (0%)	15 van de 100 patiënten (15%)	
Dagelijks functioneren na drie maanden	Net merkbare verslechtering	Net merkbare verbetering	
Buikklachten na drie maanden	Net merkbare afname	Net merkbare afname	
Levensverwachting	9 maanden	9 maanden	

I choose: Treatment A / Treatment B / Basic care
(circle your choice)

Choice task 4
Could you indicate if you would choose for **Treatment A, Treatment B** or **Basic care:**

	**Behandeling A**	**Behandeling B**	**Basiszorg**
Aantal ziekenhuisbezoeken	3 bezoeken per 4 weken<br>MA DI WO DO VR ZA ZO<br>DO: X, X, X	1 bezoek per 4 weken<br>MA DI WO DO VR ZA ZO<br>WO: X	- Geen behandeling die de kanker weghaalt of kleiner maakt.<br>- Alleen afspraken in het ziekenhuis bij klachten.<br>- Door de basiszorg heeft u minder klachten en u voelt zich wat beter.<br>- De helft van de patiënten die alleen basiszorg krijgt, leeft korter dan twee maanden. De andere helft leeft langer.
Kans op een extra ziekenhuisopname	30 van de 100 patiënten (30%)<br>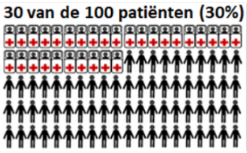	15 van de 100 patiënten (15%)<br>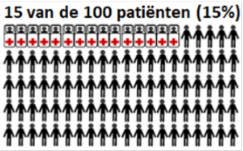	
Dagelijks functioneren na drie maanden	Geen verschil	Net merkbare verslechtering	
Buikklachten na drie maanden	Geen verschil	Duidelijk merkbare afname	
Levensverwachting	**12 maanden**	**9 maanden**	

I choose: Treatment A / Treatment B / Basic care
(circle your choice)

Choice task 5
Could you indicate if you would choose for **Treatment A, Treatment B** or **Basic care:**

	**Behandeling A**	**Behandeling B**	**Basiszorg**
Aantal ziekenhuisbezoeken	3 bezoeken per 4 weken<br>MA DI WO DO VR ZA ZO<br>DO: X, X, X	1 bezoek per 4 weken<br>MA DI WO DO VR ZA ZO<br>WO: X	- Geen behandeling die de kanker weghaalt of kleiner maakt.<br>- Alleen afspraken in het ziekenhuis bij klachten.<br>- Door de basiszorg heeft u minder klachten en u voelt zich wat beter.<br>- De helft van de patiënten die alleen basiszorg krijgt, leeft korter dan twee maanden. De andere helft leeft langer.
Kans op een extra ziekenhuisopname	15 van de 100 patiënten (15%)<br>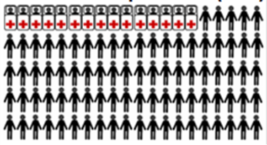	30 van de 100 patiënten (30%)<br>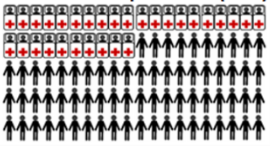	
Dagelijks functioneren na drie maanden	Geen verschil	Net merkbare verbetering	
Buikklachten na drie maanden	Geen verschil	Net merkbare afname	
Levensverwachting	**9 maanden**	**12 maanden**	

I choose: Treatment A / Treatment B / Basic care
(circle your choice)

Choice task 6
Could you indicate if you would choose for **Treatment A, Treatment B** or **Basic care:**

	**Behandeling A**	**Behandeling B**	**Basiszorg**
Aantal ziekenhuisbezoeken	**1 bezoek per 4 weken**<br>MA DI WO DO VR ZA ZO<br>X (DO)	**2 bezoeken per 4 weken**<br>MA DI WO DO VR ZA ZO<br>X (DO)<br>X (DO)	- Geen behandeling die de kanker weghaalt of kleiner maakt.<br>- Alleen afspraken in het ziekenhuis bij klachten.<br>- Door de basiszorg heeft u minder klachten en u voelt zich wat beter.<br>- De helft van de patiënten die alleen basiszorg krijgt, leeft korter dan twee maanden. De andere helft leeft langer.
Kans op een extra ziekenhuisopname	**30 van de 100 patiënten (30%)**<br>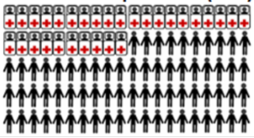	**0 van de 100 patiënten (0%)**<br>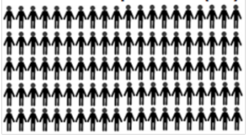	
Dagelijks functioneren na drie maanden	**Net merkbare verslechtering**	**Geen verschil**	
Buikklachten na drie maanden	**Geen verschil**	**Net merkbare toename**	
Levensverwachting	**6 maanden**	**9 maanden**	

I choose: Treatment A / Treatment B / Basic care
(circle your choice)

Choice task 7
Could you indicate if you would choose for **Treatment A, Treatment B** or **Basic care:**

	**Behandeling A**	**Behandeling B**	**Basiszorg**
Aantal ziekenhuisbezoeken	**1 bezoek per 4 weken**<br>MA DI WO DO VR ZA ZO<br>X (DO)	**3 bezoeken per 4 weken**<br>MA DI WO DO VR ZA ZO<br>X (DO)<br>X (DO)<br>X (DO)	- Geen behandeling die de kanker weghaalt of kleiner maakt.<br>- Alleen afspraken in het ziekenhuis bij klachten.<br>- Door de basiszorg heeft u minder klachten en u voelt zich wat beter.<br>- De helft van de patiënten die alleen basiszorg krijgt, leeft korter dan twee maanden. De andere helft leeft langer.
Kans op een extra ziekenhuisopname	**15 van de 100 patiënten (15%)**<br>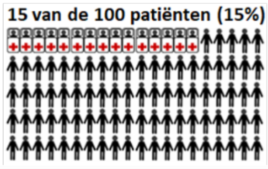	**0 van de 100 patiënten (0%)**<br>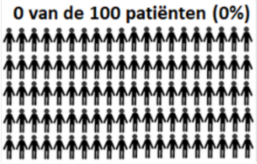	
Dagelijks functioneren na drie maanden	**Net merkbare verbetering**	**Net merkbare verslechtering**	
Buikklachten na drie maanden	**Geen verschil**	**Duidelijk merkbare afname**	
Levensverwachting	**6 maanden**	**3 maanden**	

I choose: Treatment A / Treatment B / Basic care
(circle your choice)

Choice task 8
Could you indicate if you would choose for **Treatment A, Treatment B** or **Basic care:**

	**Behandeling A**	**Behandeling B**	**Basiszorg**
Aantal ziekenhuisbezoeken	**3 bezoeken per 4 weken**<br>MA DI WO DO VR ZA ZO<br>DO: X, X, X	**2 bezoeken per 4 weken**<br>MA DI WO DO VR ZA ZO<br>DO: X, X	- Geen behandeling die de kanker weghaalt of kleiner maakt.<br>- Alleen afspraken in het ziekenhuis bij klachten.<br>- Door de basiszorg heeft u minder klachten en u voelt zich wat beter.<br>- De helft van de patiënten die alleen basiszorg krijgt, leeft korter dan twee maanden. De andere helft leeft langer.
Kans op een extra ziekenhuisopname	**0 van de 100 patiënten (0%)**<br>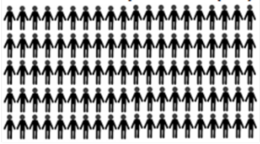	**30 van de 100 patiënten (30%)**<br>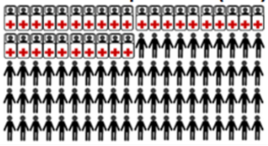	
Dagelijks functioneren na drie maanden	**Net merkbare verbetering** ↑	**Net merkbare verslechtering** ↓	
Buikklachten na drie maanden	**Duidelijk merkbare afname** ↓↓	**Net merkbare toename** ↑	
Levensverwachting	**3 maanden**	**9 maanden**	

I choose: Treatment A / Treatment B / Basic care
(circle your choice)

Choice task 9
Could you indicate if you would choose for **Treatment A, Treatment B** or **Basic care:**

	**Behandeling A**	**Behandeling B**	**Basiszorg**
Aantal ziekenhuisbezoeken	**2 bezoeken per 4 weken**<br>MA DI WO DO VR ZA ZO<br>DO: X, X	**3 bezoeken per 4 weken**<br>MA DI WO DO VR ZA ZO<br>DO: X, X, X	- Geen behandeling die de kanker weghaalt of kleiner maakt.<br>- Alleen afspraken in het ziekenhuis bij klachten.<br>- Door de basiszorg heeft u minder klachten en u voelt zich wat beter.<br>- De helft van de patiënten die alleen basiszorg krijgt, leeft korter dan twee maanden. De andere helft leeft langer.
Kans op een extra ziekenhuisopname	**0 van de 100 patiënten (0%)**<br>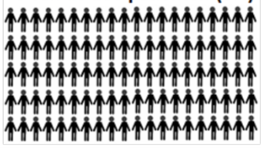	**30 van de 100 patiënten (30%)**<br>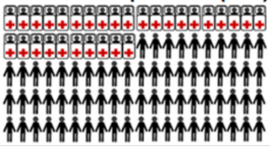	
Dagelijks functioneren na drie maanden	**Net merkbare verbetering** ↑	**Geen verschil** ●	
Buikklachten na drie maanden	**Net merkbare afname** ↓	**Geen verschil** ●	
Levensverwachting	**3 maanden**	**9 maanden**	

I choose: Treatment A / Treatment B / Basic care
(circle your choice)

Choice task 10
Could you indicate if you would choose for **Treatment A, Treatment B** or **Basic care:**

	**Behandeling A**	**Behandeling B**	**Basiszorg**
Aantal ziekenhuisbezoeken	1 bezoek per 4 weken<br>MA DI WO DO VR ZA ZO<br>X	2 bezoeken per 4 weken<br>MA DI WO DO VR ZA ZO<br>X<br>X	- Geen behandeling die de kanker weghaalt of kleiner maakt.<br>- Alleen afspraken in het ziekenhuis bij klachten.<br>- Door de basiszorg heeft u minder klachten en u voelt zich wat beter.<br>- De helft van de patiënten die alleen basiszorg krijgt, leeft korter dan twee maanden. De andere helft leeft langer.
Kans op een extra ziekenhuisopname	0 van de 100 patiënten (0%)	30 van de 100 patiënten (30%)	
Dagelijks functioneren na drie maanden	Geen verschil	Net merkbare verbetering	
Buikklachten na drie maanden	Net merkbare afname	Duidelijk merkbare afname	
Levensverwachting	**12 maanden**	**12 maanden**	

I choose: Treatment A / Treatment B / Basic care
(circle your choice)

Choice task 11
Could you indicate if you would choose for **Treatment A, Treatment B** or **Basic care:**

	**Behandeling A**	**Behandeling B**	**Basiszorg**
Aantal ziekenhuisbezoeken	1 bezoek per 4 weken<br>MA DI WO DO VR ZA ZO<br>X	2 bezoeken per 4 weken<br>MA DI WO DO VR ZA ZO<br>X<br>X	- Geen behandeling die de kanker weghaalt of kleiner maakt.<br>- Alleen afspraken in het ziekenhuis bij klachten.<br>- Door de basiszorg heeft u minder klachten en u voelt zich wat beter.<br>- De helft van de patiënten die alleen basiszorg krijgt, leeft korter dan twee maanden. De andere helft leeft langer.
Kans op een extra ziekenhuisopname	30 van de 100 patiënten (30%)	15 van de 100 patiënten (15%)	
Dagelijks functioneren na drie maanden	Geen verschil	Net merkbare verslechtering	
Buikklachten na drie maanden	Net merkbare afname	Geen verschil	
Levensverwachting	**6 maanden**	**3 maanden**	

I choose: Treatment A / Treatment B / Basic care
(circle your choice)

Choice task 12
Could you indicate if you would choose for **Treatment A, Treatment B** or **Basic care:**

	Behandeling A	Behandeling B	Basiszorg
Aantal ziekenhuisbezoeken	1 bezoek per 4 weken<br>MA DI WO DO VR ZA ZO<br>X	3 bezoeken per 4 weken<br>MA DI WO DO VR ZA ZO<br>X<br>X<br>X	- Geen behandeling die de kanker weghaalt of kleiner maakt.<br>- Alleen afspraken in het ziekenhuis bij klachten.<br>- Door de basiszorg heeft u minder klachten en u voelt zich wat beter.<br>- De helft van de patiënten die alleen basiszorg krijgt, leeft korter dan twee maanden. De andere helft leeft langer.
Kans op een extra ziekenhuisopname	15 van de 100 patiënten (15%)	0 van de 100 patiënten (0%)	
Dagelijks functioneren na drie maanden	Net merkbare verslechtering	Net merkbare verbetering	
Buikklachten na drie maanden	Net merkbare toename	Geen verschil	
Levensverwachting	6 maanden	6 maanden	

I choose: Treatment A / Treatment B / Basic care
(circle your choice)

Part C: What is important to you?

You have now completed the twelve choice tasks. In these assignments we asked you to choose an imaginary treatment based on five characteristics. Can you give these characteristics a number from 1 to 5 below, where 1 stands for “most important” and 5 stands for “least important”?

Characteristic	Number
Number of hospital visits	
Chance for an extra hospital referral	
Daily functioning after three months	
Abdominal complaints after three months	
Life expectancy	

We study when patients would choose a certain treatment. Did you miss something in this questionnaire? For example, a characteristic to describe a treatment?

O I haven’t missed anything
O I have missed something, which is:

Part C: Feedback

We now ask you your opinion about the survey.

Was it hard for you **to understand** the survey?

- O It was not hard for me at all to understand the survey.
- O It was not hard for me to understand the survey.
- O It was a little hard for me to understand the survey.
- O It was hard for me to understand the survey.
- O It was very hard for me to understand the survey.

Was it hard for you **to fill in** the survey?

- O It was not hard for me at all to fill in the survey.
- O It was not hard for me to fill in the survey.
- O It was a little hard for me to fill in the survey.
- O It was hard for me to fill in the survey.
- O It was very hard for me to fill in the survey.

Do you want to give other feedback about the survey you just completed?

- O I don't want to give other feedback.
- O I want to give feedback, which is:

Do you have any further questions? If this questionnaire raises questions about your own treatment or illness, we advise you to contact your own doctor.

- O I don't have questions about this survey.
- O I have a question about this survey, which is:

May you be approached in the future for other scientific research on this subject?

- O yes
- O no

Thank you very much for your contribution!
