# Supplementary material for: Patient preferences for pancreatic cancer treatment (PERSEUS): a multicenter discrete choice experiment
Source: Health Qual Life Outcomes. 2025 Dec 24;23:122. doi: 10.1186/s12955-025-02440-5 (PMC12729412; doi:10.1186/s12955-025-02440-5)
Supplement: Supplementary file 2 — Supplementary Material 2 [file 12955_2025_2440_MOESM2_ESM.docx]

**SUPPLEMENTAL METHODS**

**Preference assessment tool**

The DCE is based on the premise that utility is derived from specific components of a good or service. Therefore, it is well-suited to evaluate the willingness of patients to tradeoff between attributes, and establish the alternative that provides the maximum utility for patients.^[[1]](#endnote-1),^^[[2]](#endnote-2)^  Furthermore, this method allows to estimate the relative strength of preferences for attributes in quantitative terms.^[[3]](#endnote-3)^ Therefore, the DCE was used to measure preferences in this project.

**Literature review**

A literature review was performed to identify patients’ preferences in pancreatic cancer treatment. It was also reported which patient-reported outcomes and quality of life aspects patients consider to be important regarding their disease and treatment. The used search term in PubMed was:

*(pancreatic neoplasm*[MeSH Terms]) AND ((patient*[MeSH Terms]) OR (family*[MeSH Terms]) OR (caregiver*[MeSH Terms]) OR (health personnel*[MeSH Terms])) AND ((quality of life*[MeSH Terms]) OR (health care outcome assessment*[MeSH Terms]) OR (Treatment adherence and compliance*[MeSH Terms])) AND ((quality of life[MeSH Terms]) OR (Treatment adherence and compliance[MesH Terms])) NOT ((neuroendocrine tumors*[MeSH Terms]) OR (islet cell adenoma*[MeSH Terms]) OR (islet cell carcinoma*[MeSH Terms]))*

The search was done in Scopus as well. In the literature screen (n=322), one researcher screened the articles while a second researcher did a quality control by screening a sample of 25% of the articles. Articles were excluded in case the article did not include pancreatic cancer, research was not performed in patients, the article was not available in English or Dutch or no full text was available, or the focus of the article was not on patient experiences or preferences regarding treatment outcomes. The papers for full text review (n=32) were divided over the two researchers. During the full-text screening data related to preferences or experiences regarding treatment outcomes were extracted, resulting in four included articles.^[[4]](#endnote-4),^^[[5]](#endnote-5),^^[[6]](#endnote-6),^^[[7]](#endnote-7)^

**Translation of EORTC QLQ-C30 scale based levels into minimal important differences**

Two DCEs were designed with different descriptions of the levels within the attributes for the early-stage disease and late-stage disease groups. Levels were selected to be realistic and reflect existing or potential treatments for pancreatic cancer, based on current guidelines, clinical trials, and observational studies that reported quality of life data according to the EORTC QLQ-C30 and PAN26 questionnaires (Supplemental Table 2). The Chance of additional hospitalization was based on the prevalence of CTCAE grade 3-4 events. The attributes and levels and their understandability were pre-tested with patients using semi-structured interviews. The attribute levels of Daily functioning and Gastrointestinal complaints, which were based on EORTC-QLQ-C30 scales and at first instance described in numerical changes (i.e. ‘your daily functioning decreases with 10 points out of 100’), were considered too complicated for the patients. Therefore, after the test phase, the descriptions ‘unnoticeable change’, ‘just noticeable change’ and ‘clearly noticeable change’ were introduced, for which the minimal important difference (MID) was used as a cut-off. How this was done, is described in the following paragraphs.

Although the EORTC is updating the values of anchor-based and distribution-based MIDs per cancer type, this has not been done yet for pancreatic cancer. Reni *et al.* studied the MIDs of PAN26 questionnaires in patients who underwent resection and showed that the anchor-based MID for gastrointestinal complaints -5.78 (the mean of MID for *digestive symptoms, bloated, indigestion, low weight* and *taste different*). The MID for *limited activities* was -8.6.^[[8]](#endnote-8)^ Musoro *et al.* analyzed the MID of several cancer types. We considered advanced colorectal cancer in patients receiving chemotherapy (with 5-FU+folinic acid+oxaliplatin; 5-FU+leucovorin; 5-FU+folinic acid+irinotecan) to be the most appropriate for comparison with pancreatic cancer. In this review, the mean MID for within-group change for daily functioning was 10.4 (improvement) and -8.1 (deterioration), and for gastrointestinal complaints it was 9.8 (improvement) and -8.1 (deterioration).^[[9]](#endnote-9)^ Therefore, we decided to use 5 as MID for gastrointestinal complaints and 9 as MID for daily functioning. Below the MID, the change in scale was considered as ‘unnoticeable’, above the MID the change was ‘just noticeable’. If the change was twice the MID, it was considered as ‘clearly noticeable’.

**Table 1. Levels for scale-based criteria based on minimal important difference**

|  | Unnoticeable | Just noticeable | Clearly noticeable | Very noticeable |
| --- | --- | --- | --- | --- |
| Daily functioning | 0-9 | 9-18 | 18-27 | >27 |
| Gastrointestinal complaints | 0-5 | 5-10 | 10-15 | >15 |

**Table 2. Literature based “middle” levels for existing treatment regimens – Daily functioning**

| **Treatment** | **Change at 3 months after baseline (QoL scale)** | **Described as** |
| --- | --- | --- |
| Surgery | Decrease 7-16 | Just noticeable |
| FOLFIRINOX (for late-stage disease) | Stabilization 0-5 | Unnoticeable |
| Gemcitabine (for late-stage disease) | Stabilization | Unnoticeable |

**Table 3. Literature based “middle” levels for existing treatment regimens – Gastrointestinal complaints**

| **Treatment** | **Change at 3 months after baseline (QoL scale)** | **Described as** |
| --- | --- | --- |
| Surgery only | Stabilization 0-5 | Unnoticeable |
| FOLFIRINOX (for late-stage disease) | Decrease 5-10 | Just noticeable |
| Gemcitabine (for late-stage disease) | Decrease 1-14 | Just noticeable |

For each attribute per treatment setting, there were 3 levels. The middle level was based on literature, and one higher and one lower level was added. For the gastrointestinal complaints in the treatment setting of late-stage disease, there were 4 levels to acknowledge the spread in the results and to add a level that described a deterioration of the gastrointestinal complaints.

**Number of hospital visits**

As for early-stage disease treatment at the moment in the Netherlands several trials are running studying the benefit of pre- and post-operative systemic treatment, we decided to pile all these regiments together in the level “surgery + 12 times a biweekly chemotherapy administration”. For the late-stage disease setting, the most common regimens (biweekly FOLFIRINOX and weekly gemcitabine + nab-paclitaxel) were used as levels.

**Attribute-ranking based prior estimation**

Attributes were ranked by five experts and patients and based on this, priors were determined. For each attribute, the lowest level was coded as a reference level, except for daily functioning and gastrointestinal complaints, for which the best effect was used as reference level. The highest prior was assigned to the longest extension of life expectancy, and the lowest prior was assigned to the hospital visits levels.

**Table 4: priors per level for the pilot phase.**

| **Early-stage disease** | | | **Late-stage disease** | | |
| --- | --- | --- | --- | --- | --- |
| **Attribute (ranking)** | **Level** | **Coefficient** | **Attribute (ranking)** | **Level** | **Coefficient** |
| Life expectancy (1) | 6 months  12 months  18 months  24 months | Reference  0.2  0.4  0.5 | Life expectancy (1) | 3 months  6 months  9 months  12 months | Reference  0.2  0.4  0.5 |
| Daily functioning (2) | Stabilization  Slight decrease  Clear decrease | Reference  -0.13  -0.25 | Daily functioning (2) | Slight increase  Stabilization  Slight decrease | Reference  -0.2  -0.4 |
| Gastrointestinal complaints (3) | Slight decrease  Stabilization  Slight increase | Reference  -0.03  -0.05 | Gastrointestinal complaints (3) | Clear decrease  Slight decrease  Stabilization  Slight increase | Reference  -0.07  -0.13  -0.25 |
| Chance for an extra hospital referral (3) | 0%  30%  60% | Reference  -0.03  -0.05 | Chance for an extra hospital referral (4) | 0%  15%  30% | Reference  -0.07  -0.13 |
| Number of hospital visits (5) | Surgery  Surgery + 6 visits  Surgery + 12 visits | Reference  -0.01  -0.03 | Number of hospital visits (5) | 1 per month  2 per month  3 per month | Reference  -0.08  -0.11 |

**Analysis of the respondent characteristics**

Continuous respondent characteristics were described by median with the minimum and maximum and interquartile range. The time in days from diagnosis to filling in the survey was defined as: The date of diagnosis was the date of the first multidisciplinary meeting where the patient was discussed. For the Amsterdam UMC included patients, the date of filling in was the date of completion, or the date of sending the survey when the survey was not completed in total. For the PACAP-included patients, the date of informed consent was used as survey-completion date.

**Equations**

Basic equation for multinomial logit model with a random effect and error:

Equation 1: *U_ijn_ = β_n_ * x_ijn_ + ε_ijn_*

Where *U* is the utility of alternative *i* for individual *n* in choice situation *j*, *x* represents a vector of attribute levels, *β* is the individual’s weight for the attribute level, and *ε_ijn_* is the error term.^[[10]](#endnote-10)^

Final model early-stage disease setting: all attributes had a significant SD and were included as random effect:

Equation 2: V = β_1_ x *Life expectancy_12_months_* + β_2_ x *Life expectancy_18_months_* + β_3_ x *Life expectancy_24_months_* + β_4_ x *Daily functioning_-9_* + β_5_ x *Daily functioning_-18_* + β_6_ x *Gastrointestinal complaints_0_* + β_7_ x *Gastrointestinal complaints_+5_* + β_8_ x *Hospital referral_30%_* + β_9_ x *Hospital referral_60%_* + β_10_ x *hospital visits_6_* + β_11_ x *hospital visits_12_*

Final model late-stage disease setting: the attributes Daily functioning and Gastrointestinal complaints did not have a significant SD and were included as fixed effects:

Equation 3: V = β_1_ x *Life expectancy_6_months_* + β_2_ x *Life expectancy_9_months_* + β_3_ x *Life expectancy_12_months_* + β_4_ x *Daily functioning_0_* + β_5_ x *Daily functioning_-9_* + β_6_ x *Gastrointestinal complaints_-5_* + β_7_ x *Gastrointestinal complaints_0_* + β_8_ x *Gastrointestinal complaints_+5_* + β_9_ x *Hospital referral_15%_* + β_10_ x *Hospital referral_30%_* + β_11_ x *hospital visits_2/month_* + β_12_ x *hospital visits_3/month_*

Equation 4: *U_1m_ = (β_life_expectancy, high_ – β_life_expectancy, ref_) / (life expectancy_high_ – life expectancy_ref_)*

Where U_1m_ is the utility of one month life extension.

Equation 5: *t_A_ = (β_A,high_ – β_A, ref_)/ U_1m_*

Where t_A_ is the minimum amount of increased life expectancy (in months) that patients would need to theoretically accept a worsening of attribute A.

**SUPPLEMENTAL REFERENCES**

1. Hauber, A.B., González, J.M., Groothuis-Oudshoorn, C.G.M., *et* *al*. (2016) Statistical Methods for the Analysis of Discrete Choice Experiments: A Report of the ISPOR Conjoint Analysis Good Research Practices Task Force. Value in Health, 19: 300-315. doi:[10.1016/j.jval.2016.04.004](https://doi.org/10.1016/j.jval.2016.04.004) [↑](#endnote-ref-1)
2. Bridges, J.F.P., Hauber, A.B., Marshall, D., *et al*. (2011) Conjoint analysis application in health - a checklist: a report of the ISPOR good research practices for conjoint analysis task force. Value Health, 14: 403-413. doi:[10.1016/j.jval.2010.11.013](https://doi.org/10.1016/j.jval.2010.11.013) [↑](#endnote-ref-2)
3. Mangham, L.J., Hanson, K., McPake, B. (2008) How to do (or not to do)… Designing a discrete choice experiment for application in low-income country. Health policy and planning, 24:151-158. doi:10.1093/heapol/czn047 [↑](#endnote-ref-3)
4. Rijssen, L., Gerritsen, A., Henselmans, I., *et al.* (2019) Core set of patient-reported outcomes in pancreatic cancer (COPRAC): An international Delphi study among patients and health care providers. Annal of Surgery, 270: 158-164. https://doi.org/10.1097/sla.0000000000002633 [↑](#endnote-ref-4)
5. Torgerson, S., Wiebe, L.A. (2013) Supportive care of the patient with advance pancreatic cancer. Oncology, 27(3). [↑](#endnote-ref-5)
6. Sun, V., Ruel, N., Chung, V., *et al*. (2016) Pilot Study of an Interdisciplinary Supportive Care Planning Intervention in Pancreatic Cancer. Support Care Cancer, 24: 3417-3424 doi: 10.1007/s00520-016-3155-9. [↑](#endnote-ref-6)
7. Molinari, M., El-Tawil, K., Swaid, F., *et al.* (2019) Patients' treatment preferences for potentially resectable tumors of the head of the pancreas. HPB, 22: 265-274. https://doi.org/10.1016/j.hpb.2019.06.015 [↑](#endnote-ref-7)
8. Reni, M. Braverman, J., Hendifar, A. *et al.* (2021) Evaluation of minimal important difference and responder definition in the EORTC QLQ-PAN26 module for assessing health-related quality of life in patients with surgically resected pancreatic adenocarcinoma. Ann Surg Oncol, 28: 7545 – 7554. https://doi.org/10.1245/s10434-021-09816-z [↑](#endnote-ref-8)
9. Musoro, J.Z., Sodergren, S.C., Coens, C. *et al.* (2020) Minimally important differences for interpreting the EORTC QLQ-C30 in advanced colorectal cancer patients treated with chemotherapy. Colorectal Disease, 22: 2278 – 2287. https://doi.org/10.1111/codi.15295 [↑](#endnote-ref-9)
10. Bao, M. Huang, C., Wang, L., Yan, G., Chen, G. (2023) Eliciting primary healthcare physician’s preferences for job characteristics in rural China: a discrete choice experiment. BMJ Open. 13 (3): e056741. https://doi.org/10.1136/bmjopen-2021-056741 [↑](#endnote-ref-10)
