# Supplementary figures and images for: Patient preferences for pancreatic cancer treatment (PERSEUS): a multicenter discrete choice experiment
Source: Health Qual Life Outcomes. 2025 Dec 24;23:122. doi: 10.1186/s12955-025-02440-5 (PMC12729412; doi:10.1186/s12955-025-02440-5)

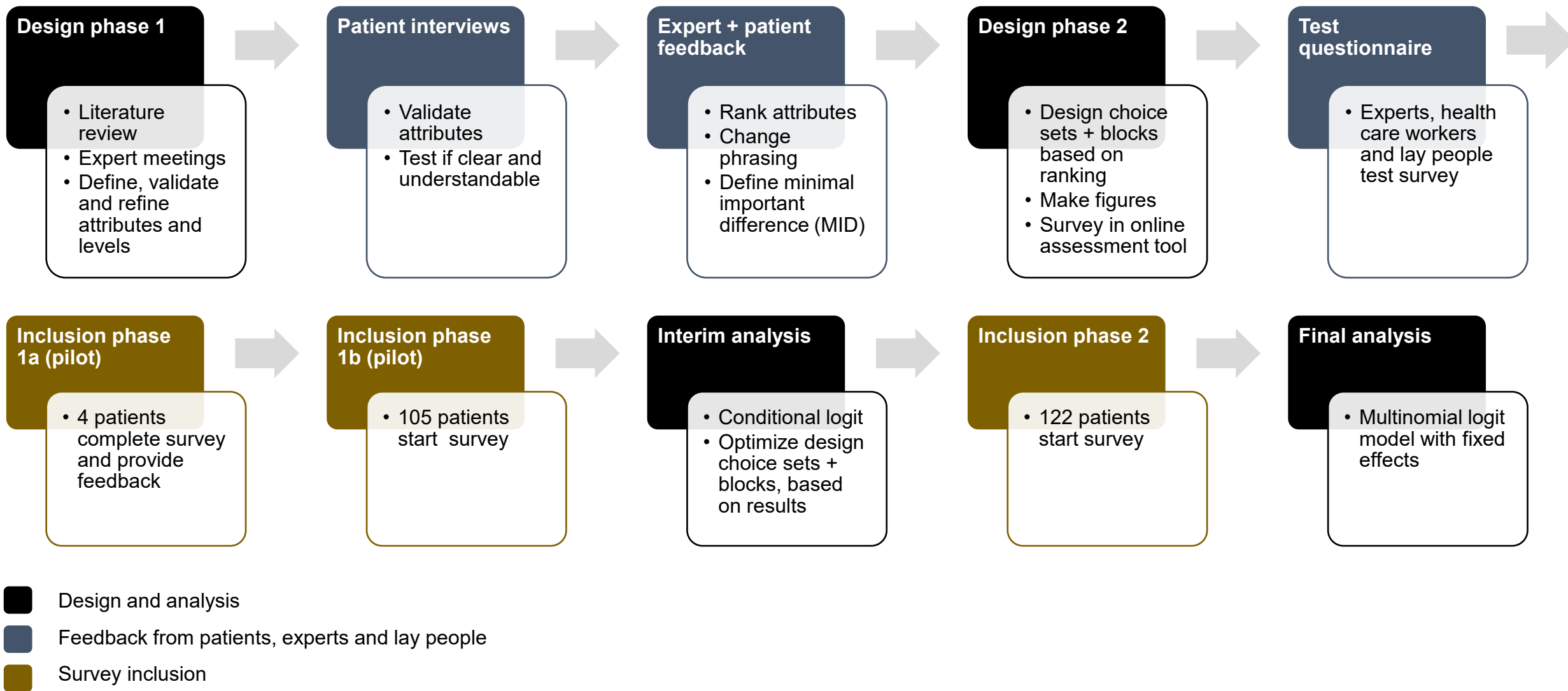

Figure S2

A

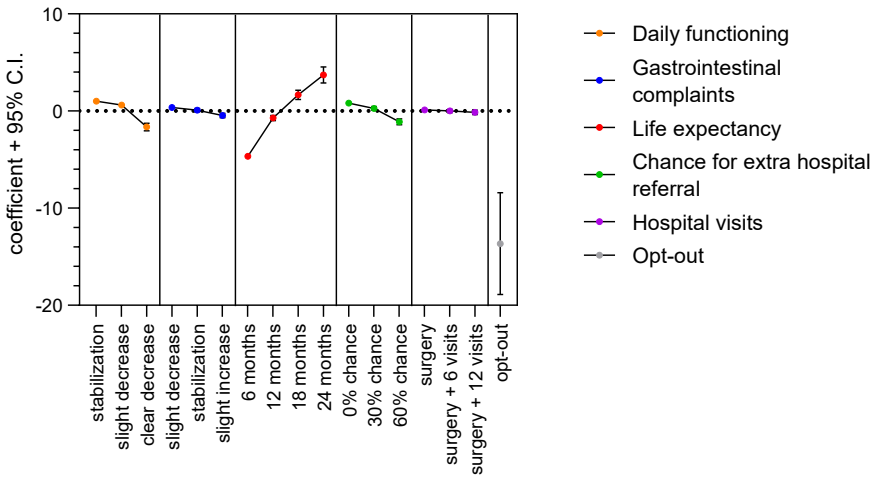

B

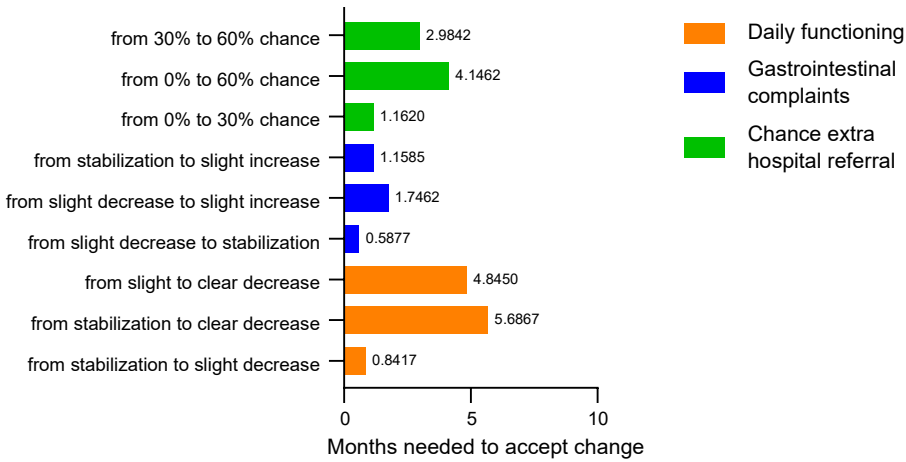

C

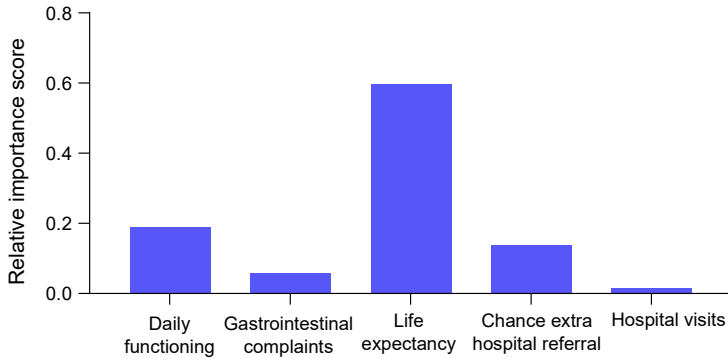

Figure S3

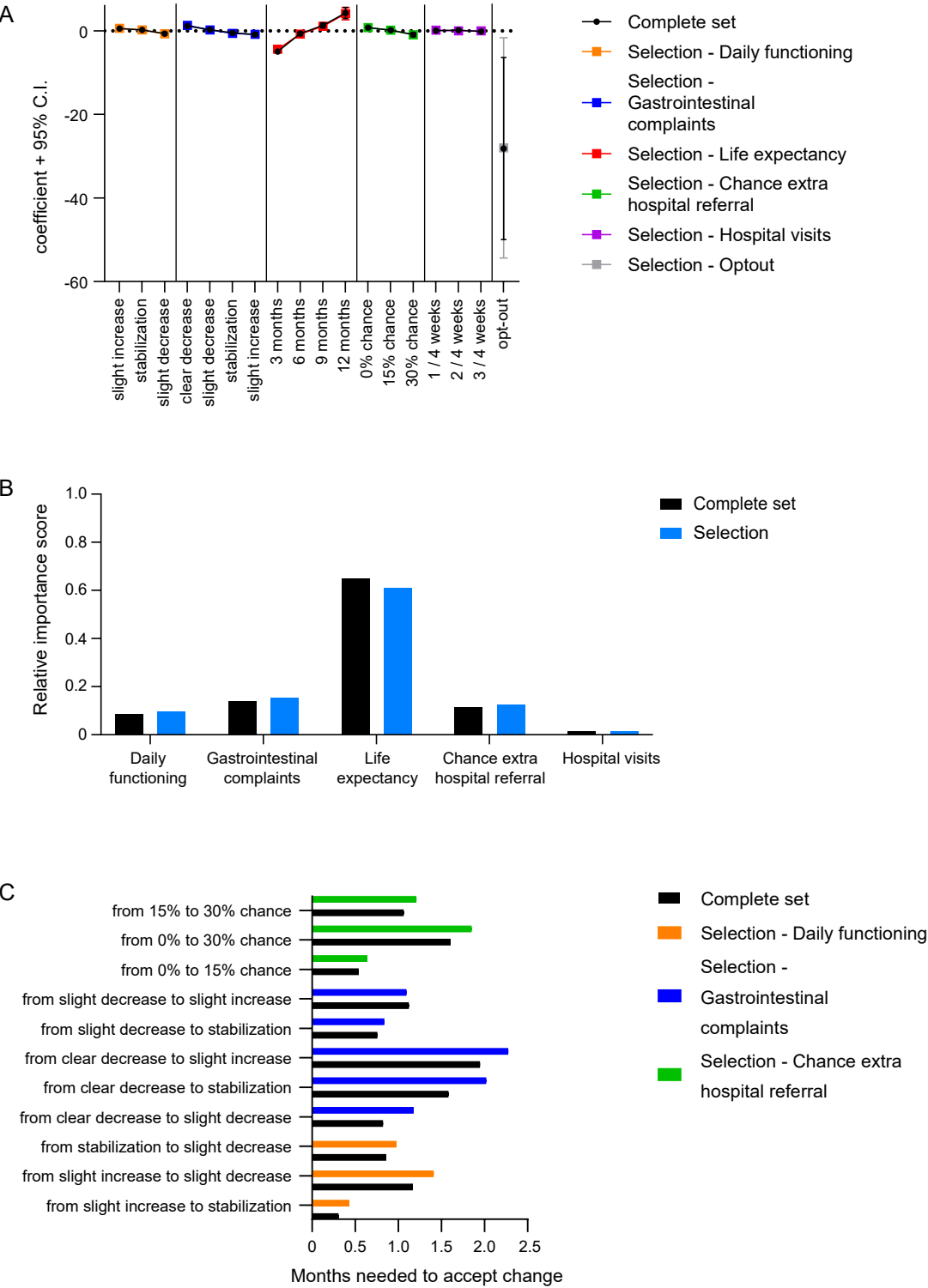

Supplement: Supplementary file 3 — Supplementary Material 3 [file 12955_2025_2440_MOESM3_ESM.pdf]
