# Supplementary material for: Patient preferences for pancreatic cancer treatment (PERSEUS): a multicenter discrete choice experiment
Source: Health Qual Life Outcomes. 2025 Dec 24;23:122. doi: 10.1186/s12955-025-02440-5 (PMC12729412; doi:10.1186/s12955-025-02440-5)
Supplement: Supplementary file 4 — Supplementary Material 4 [file 12955_2025_2440_MOESM4_ESM.docx]

**SUPPLEMENTAL TABLES**

**Supplemental Table 1 and 2:** see submitted Excel file for data.

**Supplemental Table 1: Literature review results for the title / abstract screening.**

**Supplemental Table 2: Literature used as input for level estimation.**

**Supplemental Table 3: Interim analysis results for early-stage disease setting after pilot phase.**

| **Attribute** | **Level** | **Coefficient** | **SE** | **z** | **p value** |
| --- | --- | --- | --- | --- | --- |
| Daily functioning | Slight decrease | -0.08624 | 0.131198 | -0.66 | 0.511 |
|  | Clear decrease | -1.65198 | 0.218289 | -7.57 | <0.001 |
| Gastro-intestinal complaints | No change | -0.03781 | 0.123569 | -0.31 | 0.76 |
|  | Slight increase | -0.46391 | 0.188113 | -2.47 | 0.014 |
| Life expectancy | 12 months | 1.58623 | 0.220937 | 7.18 | <0.001 |
|  | 18 months | 2.468801 | 0.337621 | 7.31 | <0.001 |
|  | 24 months | 3.611948 | 0.466844 | 7.74 | <0.001 |
| Adverse events | 30% of the patients | -0.38112 | 0.139371 | -2.73 | 0.006 |
|  | 60% of the patients | -0.81504 | 0.180432 | -4.52 | <0.001 |
| Hospital visits | 10 days hospital stay + 6 visits | -0.01346 | 0.186164 | -0.07 | 0.942 |
|  | 10 days hospital stay + 12 visits | 0.019936 | 0.196045 | 0.1 | 0.919 |
| Best supportive care | | -1.87079 | 0.702014 | -2.66 | 0.008 |

Conditional logit model results are printed for the started surveys in the early-stage disease setting (n = 64 respondents and 2100 observation). Reference level coefficients are equal to 0. SE = standard error.

**Supplemental Table 4: Interim analysis results for late-stage disease setting after pilot phase.**

| **Attribute** | **Level** | **Coefficient** | **SE** | **z** | **p value** |
| --- | --- | --- | --- | --- | --- |
| Daily functioning | Slight decrease | -0.38413 | 0.19808 | -1.94 | 0.052 |
|  | No change | -0.08015 | 0.194428 | -0.41 | 0.68 |
| Gastro-intestinal complaints | Slight decrease | -0.59573 | 0.293528 | -2.03 | 0.042 |
|  | No change | -0.82088 | 0.204956 | -4.01 | <0.001 |
|  | Slight increase | -0.76106 | 0.291165 | -2.61 | 0.009 |
| Life expectancy | 6 months | 1.777749 | 0.34918 | 5.09 | <0.001 |
|  | 9 months | 2.563946 | 0.44087 | 5.82 | <0.001 |
|  | 12 months | 3.738377 | 0.602136 | 6.21 | <0.001 |
| Adverse events | 15% of the patients | -0.26928 | 0.203886 | -1.32 | 0.187 |
|  | 30% of the patients | -0.70487 | 0.303384 | -2.32 | 0.02 |
| Hospital visits | 2 visits per 4 weeks | 0.121833 | 0.168289 | 0.72 | 0.469 |
|  | 3 visits per 4 weeks | -0.07488 | 0.177046 | -0.42 | 0.672 |
| Best supportive care | | -0.82678 | 0.667981 | -1.24 | 0.216 |

Conditional logit model results are printed for the started surveys in the late-stage disease setting (n = 45 respondents and 1638 observations). Reference level coefficients are equal to 0. SE = standard error.

**Supplemental Table 5: Attribute-level estimated for the mixed effect logit model in the early-stage disease group after exclusion of 8 patients.**

| **Attribute** | **Level** |  | **Coefficient** | **SE** | **z** | **p value** |
| --- | --- | --- | --- | --- | --- | --- |
| Daily functioning at three months after surgery | No change (reference level) | Mean | 1.003378 |  |  |  |
|  |  | SD | 0.615516 |  |  |  |
|  | Slight decrease | Mean | 0.611117 | 0.104077 | 5.87 | <0.001 |
|  |  | SD | 0.000244 | 0.153007 | 0 | 0.999 |
|  | Clear decrease | Mean | -1.64685 | 0.195482 | -8.42 | <0.001 |
|  |  | SD | 0.833339 | 0.148627 | 5.61 | <0.001 |
| Gastro-intestinal complaints at three months after surgery | Slight decrease (reference level) | Mean | 0.357523 |  |  |  |
|  |  | SD | 0.366253 |  |  |  |
|  | No change | Mean | 0.083619 | 0.086019 | 0.97 | 0.331 |
|  |  | SD | -0.03564 | 0.179368 | -0.2 | 0.842 |
|  | Slight increase | Mean | -0.4563 | 0.11763 | -3.88 | <0.001 |
|  |  | SD | 0.588702 | 0.142605 | 4.13 | <0.001 |
| Life expectancy after start treatment | 6 months (reference level) | Mean | -4.66636 |  |  |  |
|  |  | SD | 1.85797 |  |  |  |
|  | 12 months | Mean | -0.71936 | 0.134264 | -5.36 | <0.001 |
|  |  | SD | -0.0757 | 0.76364 | -0.1 | 0.921 |
|  | 18 months | Mean | 1.65351 | 0.241143 | 6.86 | <0.001 |
|  |  | SD | 1.267539 | 0.268522 | 4.72 | <0.001 |
|  | 24 months | Mean | 3.722341 | 0.419716 | 8.87 | <0.001 |
|  |  | SD | 2.185817 | 0.380584 | 5.74 | <0.001 |
| Adverse events that cause hospitalization | 0% of the patients (reference level) | Mean | 0.815956 |  |  |  |
|  |  | SD | 0.445258 |  |  |  |
|  | 30% of the patients | Mean | 0.27444 | 0.091168 | 3.01 | 0.003 |
|  |  | SD | 0.036939 | 0.168524 | 0.22 | 0.827 |
|  | 60% of the patients | Mean | -1.11633 | 0.153783 | -7.26 | <0.001 |
|  |  | SD | 0.630596 | 0.139832 | 4.51 | <0.001 |
| Number of hospital visits | 10 days hospital stay (reference level) | Mean | 0.105401 |  |  |  |
|  |  | SD | 0.466752 |  |  |  |
|  | 10 days hospital stay + 6 biweekly visits | Mean | 0.002991 | 0.087622 | 0.03 | 0.973 |
|  |  | SD | 0.212699 | 0.226255 | 0.94 | 0.347 |
|  | 10 days hospital stay + 12 biweekly visits | Mean | -0.13754 | 0.114672 | -1.2 | 0.23 |
|  |  | SD | 0.73084 | 0.141681 | 5.16 | <0.001 |
| Best supportive care | | Mean | -13.6606 | 2.671591 | -5.11 | <0.001 |
|  |  | SD | 10.1852 | 2.294193 | 4.44 | <0.001 |

Patients who did not undergo surgery or were known to have disease recurrence in the follow-up were excluded from the analysis (n = 8). Negative coefficients for the mean indicate a negative preference for the attribute level, while positive coefficients indicate a positive preference. If not significant, the level had no effect on decision-making. A significant SD indicates significant heterogeneity regarding preferences between patients. the sign of the SD is not relevant can be interpret a s positive. SE = standard error, SD = standard deviation.

**Supplemental Table 6:** **Attribute-level estimated for the mixed effect logit model with all attributes as mixed effects in the late-stage disease group after exclusion of 15 patients.**

| **Attribute** | **Level** |  | **Coefficient** | **SE** | **z** | **p value** |
| --- | --- | --- | --- | --- | --- | --- |
| Daily functioning three months after start chemotherapy | Slight increase (reference level) | Mean | 38.80228 |  |  |  |
|  |  | SD | 23.77232 |  |  |  |
|  | No change | Mean | 10.27715 | 3.50762 | 2.93 | 0.003 |
|  |  | SD | 25.88409 | 2.385265 | 10.9 | <0.001 |
|  | Slight decrease | Mean | -48.6219 | 2.785 | -17.5 | <0.001 |
|  |  | SD | 9.118381 |  |  |  |
| Gastro-intestinal complaints three months after start chemotherapy | Clear decrease (reference level) | Mean | 54.21285 |  |  |  |
|  |  | SD | 67.60906 |  |  |  |
|  | Slight decrease | Mean | 10.19807 |  |  |  |
|  |  | SD | -2.20495 | 2.471303 | -0.89 | 0.372 |
|  | No change | Mean | -26.4281 | 5.950092 | -4.44 | <0.001 |
|  |  | SD | -40.6371 | 2.994524 | -13.6 | <0.001 |
|  | Slight increase | Mean | -34.2082 | 3.043105 | -11.2 | <0.001 |
|  |  | SD | 63.04871 | 3.087031 | 20.4 | <0.001 |
| Life expectancy after start treatment | 3 months (reference level) | Mean | -246.497 |  |  |  |
|  |  | SD | 124.4203 |  |  |  |
|  | 6 months | Mean | -32.6645 | 4.229911 | -7.72 | <0.001 |
|  |  | SD | -24.0492 | 6.892383 | -3.49 | <0.001 |
|  | 9 months | Mean | 59.21358 | 6.073506 | 9.75 | <0.001 |
|  |  | SD | 41.73836 |  |  |  |
|  | 12 months | Mean | 158.2941 |  |  |  |
|  |  | SD | 133.2609 | 4.682002 | 28.5 | <0.001 |
| Adverse events that cause hospitalization | 0% of the patients (reference level) | Mean | 31.81246 |  |  |  |
|  |  | SD | 76.08686 |  |  |  |
|  | 15% of the patients | Mean | 17.63212 | 3.290035 | 5.36 | <0.001 |
|  |  | SD | -25.98 |  |  |  |
|  | 30% of the patients | Mean | -53.1955 | 4.617699 | -11.5 | <0.001 |
|  |  | SD | 82.18478 | 3.826268 | 21.5 | <0.001 |
| Number of hospital visits | 1 visit per 4 weeks (reference level) | Mean | 14.66282 |  |  |  |
|  |  | SD | 49.0288 |  |  |  |
|  | 2 visits per 4 weeks | Mean | -13.0519 |  |  |  |
|  |  | SD | 33.56884 | 2.48356 | 13.5 | <0.001 |
|  | 3 visits per 4 weeks | Mean | -5.45158 | 6.766666 | -0.81 | 0.42 |
|  |  | SD | -61.0665 | 6.012534 | -10.2 | <0.001 |
| Best supportive care | | Mean | -490.696 | 66.7192 | -7.35 | <0.001 |
|  |  | SD | 455.7037 | 73.00549 | 6.24 | <0.001 |

Patients who underwent surgery and had no known recurrence in the follow-up were excluded from the analysis (n = 15). Model resulting from 13690 Halton draws instead of 14000. Negative coefficients for the mean indicate a negative preference for the attribute level, while positive coefficients indicate a positive preference. If not significant, the level had no effect on decision-making. A significant SD indicates significant heterogeneity regarding preferences between patients. the sign of the SD is not relevant can be interpret a s positive. SE = standard error, SD = standard deviation.

**Supplemental Table 7:** **Attribute-level estimated for the mixed effect logit model with Life expectancy, Adverse events and Hospital visits as mixed effects in the late-stage disease group after exclusion of 15 patients.**

| **Attribute** | **Level** |  | **Coefficient** | **SE** | **z** | **p value** |
| --- | --- | --- | --- | --- | --- | --- |
| Daily functioning three months after start chemotherapy | Slight increase (reference level) | Mean | 0.579346 |  |  |  |
|  | No change | Mean | 0.171684 | 0.175095 | 0.98 | 0.327 |
|  | Slight decrease | Mean | -0.75103 | 0.200945 | -3.74 | <0.001 |
| Gastro-intestinal complaints three months after start chemotherapy | Clear decrease (reference level) | Mean | 1.291649 |  |  |  |
|  | Slight decrease | Mean | 0.17785 | 0.224203 | 0.79 | 0.428 |
|  | No change | Mean | -0.61297 | 0.222433 | -2.76 | 0.006 |
|  | Slight increase | Mean | -0.85653 | 0.218882 | -3.91 | <0.001 |
| Life expectancy after start treatment | 3 months (reference level) | Mean | -4.40208 |  |  |  |
|  |  | SD | 1.92553 |  |  |  |
|  | 6 months | Mean | -0.80359 | 0.26896 | -2.99 | 0.003 |
|  |  | SD | 0.403383 | 0.422007 | 0.96 | 0.339 |
|  | 9 months | Mean | 1.102867 | 0.282256 | 3.91 | <0.001 |
|  |  | SD | -0.09064 | 0.898948 | -0.1 | 0.92 |
|  | 12 months | Mean | 4.088021 | 0.758627 | 5.39 | <0.001 |
|  |  | SD | 2.578683 | 0.667523 | 3.86 | <0.001 |
| Adverse events that cause hospitalization | 0% of the patients (reference level) | Mean | 0.764497 |  |  |  |
|  |  | SD | 0.974396 |  |  |  |
|  | 15% of the patient | Mean | 0.159945 | 0.181146 | 0.88 | 0.377 |
|  |  | SD | -0.36047 | 0.341552 | -1.06 | 0.291 |
|  | 30% of the patients | Mean | -0.98127 | 0.287104 | -3.42 | 0.001 |
|  |  | SD | 1.296153 | 0.30003 | 4.32 | <0.001 |
| Number of hospital visits | 1 visit per 4 weeks (reference level) | Mean | 0.157806 |  |  |  |
|  |  | SD | 0.553941 |  |  |  |
|  | 2 visits per 4 weeks | Mean | -0.04309 | 0.165481 | -0.26 | 0.795 |
|  |  | SD | 0.002454 | 0.343971 | 0.01 | 0.994 |
|  | 3 visits per 4 weeks | Mean | -0.04993 | 0.206078 | -0.24 | 0.809 |
|  |  | SD | -0.79676 | 0.254869 | -3.13 | 0.002 |
| Best supportive care | | Mean | -28.0173 | 13.45249 | -2.08 | 0.037 |
|  |  | SD | 21.97556 | 11.34467 | 1.94 | 0.053 |

Patients who underwent surgery and had no known recurrence in the follow-up were excluded from the analysis (n = 15). In the model, Daily functioning and Gastro-intestinal complaints were treated as fixed effects and Life expectancy, Adverse events, and Hospital visits were treated as mixed effects. Negative coefficients for the mean indicate a negative preference for the attribute level, while positive coefficients indicate a positive preference. If not significant, the level had no effect on decision-making. A significant SD indicates significant heterogeneity regarding preferences between patients. the sign of the SD is not relevant can be interpret a s positive. SE = standard error, SD = standard deviation.
