# Supplementary material for: Patient preferences for pancreatic cancer treatment (PERSEUS): a multicenter discrete choice experiment
Source: Health Qual Life Outcomes. 2025 Dec 24;23:122. doi: 10.1186/s12955-025-02440-5 (PMC12729412; doi:10.1186/s12955-025-02440-5)
Supplement: Supplementary file 6 — Supplementary Material 6 [file 12955_2025_2440_MOESM6_ESM.docx]

**Supplemental Table 1: Literature review results for the title / abstract screening.**

**Supplemental Table 2: Literature used as input for level estimation.**

**Supplemental Table 3: Interim analysis results for early-stage disease setting after pilot phase.** Conditional logit model results are printed for the started surveys in the early-stage disease setting (n = 64 respondents and 2100 observations). Reference level coefficients are equal to 0. SE = standard error.

**Supplemental Table 4: Interim analysis results for late-stage disease setting after pilot phase.** Conditional logit model results are printed for the started surveys in the late-stage disease setting (n = 45 respondents and 1638 observations). Reference level coefficients are equal to 0. SE = standard error.

**Supplemental Table 5: Attribute-level estimated for the mixed effect logit model in the early-stage disease group after exclusion of 8 patients.** Patients who did not undergo surgery or were known to have disease recurrence in the follow-up were excluded from the analysis (n = 8). Negative coefficients for the mean indicate a negative preference for the attribute level, while positive coefficients indicate a positive preference. If not significant, the level had no effect on decision-making. A significant SD indicates significant heterogeneity regarding preferences between patients. the sign of the SD is not relevant can be interpret a s positive. SE = standard error, SD = standard deviation.

**Supplemental Table 6:** **Attribute-level estimated for the mixed effect logit model with all attributes as mixed effects in the late-stage disease group after exclusion of 15 patients.** Patients who underwent surgery and had no known recurrence in the follow-up were excluded from the analysis (n = 15). Model resulting from 13690 Halton draws instead of 14000. Negative coefficients for the mean indicate a negative preference for the attribute level, while positive coefficients indicate a positive preference. If not significant, the level had no effect on decision-making. A significant SD indicates significant heterogeneity regarding preferences between patients. the sign of the SD is not relevant can be interpret a s positive. SE = standard error, SD = standard deviation.

**Supplemental Table 7: Attribute-level estimated for the mixed effect logit model with Life expectancy, Adverse events and Hospital visits as mixed effects in the late-stage disease group after exclusion of 15 patients.** Patients who underwent surgery and had no known recurrence in the follow-up were excluded from the analysis (n = 15). In the model, Daily functioning and Gastro-intestinal complaints were treated as fixed effects and Life expectancy, Adverse events, and Hospital visits were treated as mixed effects. Negative coefficients for the mean indicate a negative preference for the attribute level, while positive coefficients indicate a positive preference. If not significant, the level had no effect on decision-making. A significant SD indicates significant heterogeneity regarding preferences between patients. the sign of the SD is not relevant can be interpret a s positive. SE = standard error, SD = standard deviation.

**Supplemental Figure 1: Overview of steps taken to design, conduct and analyse the PERSEUS study.**

**Supplemental Figure 2: Mixed-model estimates in early-stage disease setting after exclusion of patients who had a recurrence or could not have surgery.** (A) Mixed-model estimates for early-stage disease setting selection. (B) Relative important scores per attributes. Scores add up to one. (C) Months of increased life expectancy needed to accept a certain level of side effects. Hospital visits were not relevant in decision-making and therefore not included. Horizontal bars depict how many months of life expectancy should be added to compensate for the utility loss caused by a change in side effects or quality of life.

**Supplemental Figure 3: Mixed-model estimates in late-stage disease setting in the complete set and after exclusion of patients who underwent surgery after neoadjuvant treatment and did not have a recurrence.** (A) Mixed-model estimates for complete late-stage disease set (black circles) and selection (coloured blocks). (B) Relative important scores per attributes and patient selection. Per selection, scores add up to one. (C) Months of increased life expectancy needed to accept a certain level of side effects. Hospital visits were not relevant in decision-making and therefore not included. Horizontal bars depict how many months of life expectancy should be added to compensate for the utility loss caused by a change in side effects or quality of life.
